# A Novel Poly(3-hexylthiophene) Engineered Interface for Electrochemical Monitoring of Ascorbic Acid During the Occurrence of Glutamate-Induced Brain Cytotoxic Edemas

**DOI:** 10.34133/research.0149

**Published:** 2023-05-23

**Authors:** Zexuan Meng, Yuchan Zhang, Lu Yang, Shuang Zhao, Qiang Zhou, Jiajia Chen, Jiuxi Sui, Jian Wang, Lizhong Guo, Luyue Chang, Jialing He, Guixue Wang, Guangchao Zang

**Affiliations:** ^1^ Institute of Life Science, and Laboratory of Tissue and Cell Biology, Lab Teaching and Management Center, Chongqing Medical University, Chongqing 400016, China.; ^2^ Key Laboratory for Biorheological Science and Technology of Ministry of Education, State and Local Joint Engineering Laboratory for Vascular Implants, Bioengineering College of Chongqing University, Chongqing 400030, China.; ^3^ Jinfeng Laboratory, Chongqing 401329, China.; ^4^ Department of Pathophysiology, Chongqing Medical University, Chongqing, China.

## Abstract

Although neuroelectrochemical sensing technology offers unique benefits for neuroscience research, its application is limited by substantial interference in complex brain environments while ensuring biosafety requirements. In this study, we introduced poly(3-hexylthiophene) (P3HT) and nitrogen-doped multiwalled carbon nanotubes (N-MWCNTs) to construct a composite membrane-modified carbon fiber microelectrode (CFME/P3HT-N-MWCNTs) for ascorbic acid (AA) detection. The microelectrode presented good linearity, selectivity, stability, antifouling, and biocompatibility and exhibited great performance for application in neuroelectrochemical sensing. Subsequently, we applied CFME/P3HT-N-MWCNTs to monitor AA release from in vitro nerve cells, ex vivo brain slices, and in vivo living rat brains and determined that glutamate can induce cell edema and AA release. We also found that glutamate activated the *N*-methyl-d-aspartic acid receptor, which enhanced Na^+^ and Cl^−^ inflow to induce osmotic stress, resulting in cytotoxic edema and ultimately AA release. This study is the first to observe the process of glutamate-induced brain cytotoxic edema with AA release and to reveal the mechanism. Our work can benefit the application of P3HT in in vivo implant microelectrode construction to monitor neurochemicals, understand the molecular basis of nervous system diseases, and discover certain biomarkers of brain diseases.

## Introduction

Electrochemical sensing techniques offer direct and quantitative monitoring of the dynamic level of chemicals and can help confirm or identify prospective chemical parameters in creature [1,2]. Thus far, neuroelectrochemical sensing technology has been extensively applied at numerous biological levels, including in vitro nerve cells, ex vivo brain slices, and in vivo biological levels [3–5]. One or more neurotransmitter markers in situ and in real time can be directly monitored by further improving the diverse performance of electrodes in neuroelectrochemical sensing technology. In neurochemical monitoring, the electrodes are surrounded by a complex physiological environment, suggesting clear requirements for electrochemical electrodes. These requirements include specific quantitative analyses of target substances and withstand contamination to guarantee the accuracy of monitoring data in the future [3–7]. During the occurrence and development of neurological diseases (e.g., Parkinson’s disease), the release of neurotransmitters is intermittent, which is an abnormal case. Long-term monitoring of these diseases is a useful method to detect their fluctuation levels. After years of research, our team determined that real-time monitoring of specific markers can provide irreplaceable tools for neuroscience from a new dimension [8,9]. Thus, electrodes should be stable, biocompatible, and resistant to harm from outside forces [10–13], and an excellent electrode modification strategy is particularly crucial.

A membrane-like material frequently serves a crucial role in biomarker monitoring by preventing external interference and transporting catalytic ingredients while ensuring biosafety [14–16]. 1,3-Phenylenediamine, ethylenedioxythiophene, and polytannic acid have been used to construct membrane materials to detect neurochemicals in the brain. 3-Hexylthiophene (3HT) is a superior membrane-forming substance that can be utilized to form poly(3HT) (P3HT) on substrate materials using slow solvent drying techniques to polymerize 3HT monomers in situ [17,18]. P3HT may increase the precision and security of in vivo sensing because it has superior electrical, mechanical, and biocompatibility qualities [19–24]. P3HT can also form a membrane on the surface of a catalytic material, resulting in tightly knit nanocrystals. Furthermore, P3HT can act as an effective charge transport pathway and boost catalytic effectiveness, thereby enhancing the detection capability of electrodes [25,26]. Given P3HT’s excellent membrane formation ability, electrical properties, mechanical properties, and biocompatibility, it can be utilized in the design of implantable materials.

Cerebral edema, a pathological marker of excitotoxic injury, is the leading cause of in-hospital death. It occurs in 60% of patients with intracranial mass lesions and 15% of patients with normal initial brain computed tomography scans [27]. Therefore, the pathophysiology, process, and techniques of illness control must be investigated. Na^+^ and Cl^−^ enter cells abnormally, and the increased intracellular osmotic pressure permits water to enter cells along a concentration gradient. Furthermore, neurons experience cytotoxic edema due to ischemia and traumatic brain injury [28]. Glutamate plays an important role in the beginning of cytotoxic edema [29,30]. In a range of physiological or pathological situations, glutamate participates in brain functions as an excitatory amino acid and as a crucial neurotransmitter in the central nervous system [31,32]. The neurotoxic effects of glutamate are specifically caused by the activation of the *N*-methyl-d-aspartic acid receptor (NMDAR). Activating NMDAR during brain injury can cause cytotoxic edema and hasten neuronal death [33]. Ascorbic acid (AA) can be released into the intercellular fluid via volume-sensitive organic anion channels (VSOACs) with the occurrence of cytotoxic edema [29,34,35]. It also has antioxidant and neuroprotective properties that can lessen neuronal damage [36,37]. AA may also promote glutamate uptake into the interior of the cell via the glutamate-AA heteroexchange process, which would diminish NMDAR activation [38,39]. However, a direct link has not yet been established between the occurrence of neuronal cytotoxic edema and the release of AA generated by glutamate upon NMDAR stimulation.

Here, a new carbon fiber microelectrode (CFME) [CFME/P3HT-nitrogen-doped multiwalled carbon nanotubes (N-MWCNTs)] is modified by a P3HT-N-MWCNT composite membrane. The membrane is prepared using a one-pot method under ambient temperature and atmospheric pressure for in situ electrochemical sensing monitoring of AA in real biological systems. We systematically evaluate the electrochemical performance and biomonitor the CFME performance. The electrode could satisfy the requirements for monitoring biological systems by achieving good linearity, selectivity, stability, antifouling, and biocompatibility. Then, CFME/P3HT-N-MWCNTs were used to monitor AA in HT22 cells, brain slices, and living rat brains. The results indicate that these materials have excellent potential for in situ tissue monitoring. Finally, we examined how brain cytotoxic edema would cause AA release using the produced sensors. We monitored the AA concentration in intercellular fluid to determine the activation and inhibition of the NMDAR and Cl^−^ channels, which proved that the process of cytotoxic edema brought on by the Na^+^ and Cl^−^ influx induced by glutamate controls AA release. Monitoring the dynamics of AA release in vitro, ex vivo, and in vivo under physiological and pathological settings by CFME/P3HT-N-MWCNTs demonstrates their practical monitoring performances and potential for mechanistic studying of neuroscience. That is, P3HT membranes exhibit great performance in constructing electrochemical electrodes for in vivo monitoring, allowing the investigation of the dynamic change of neurotransmitters and the basic characteristics of nervous system diseases.

## Results

### Preparation and physical characterization of CFME/P3HT-N-MWCNTs

**Fig. 1.**
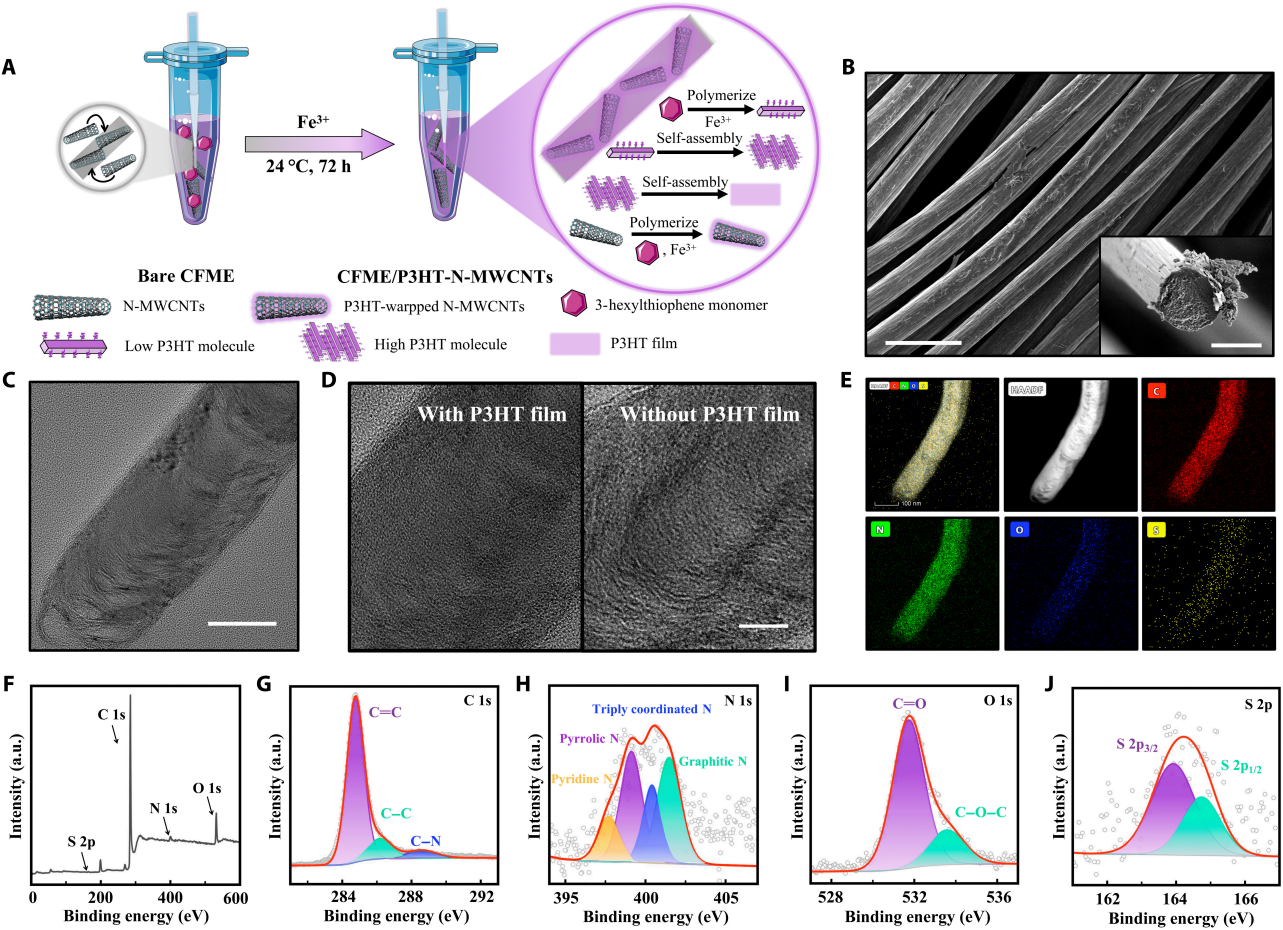
Preparation and physical characterization of CFME/P3HT-N-MWCNTs. (A) Schematic of the fabrication of a CFME/P3HT-N-MWCNTs. (B) SEM images of the CFME/P3HT-N-MWCNTs. Scale bar, 25 μm. Inset: The cross-sectional view of electrode. Scale bar, 5 μm. (C) TEM image of a P3HT-wrapped N-MWCNT. Scale bar, 50 nm. (D) TEM image of magnified N-MWCNTs with P3HT film (left) and without P3HT film (right). Scale bar, 20 nm. (E) High-angle annular dark-field (HAADF) STEM image and STEM-EDS elemental mapping images of P3HT wrapped N-MWCNT. Scale bar, 100 nm. (F) XPS survey of CFME/P3HT-N-MWCNTs. XPS spectra of C 1s (G), N 1s (H), O 1s (I), and S 2p (J) of CFME/P3HT-N-MWCNTs. a.u., arbitrary units.

**Fig. 2.**
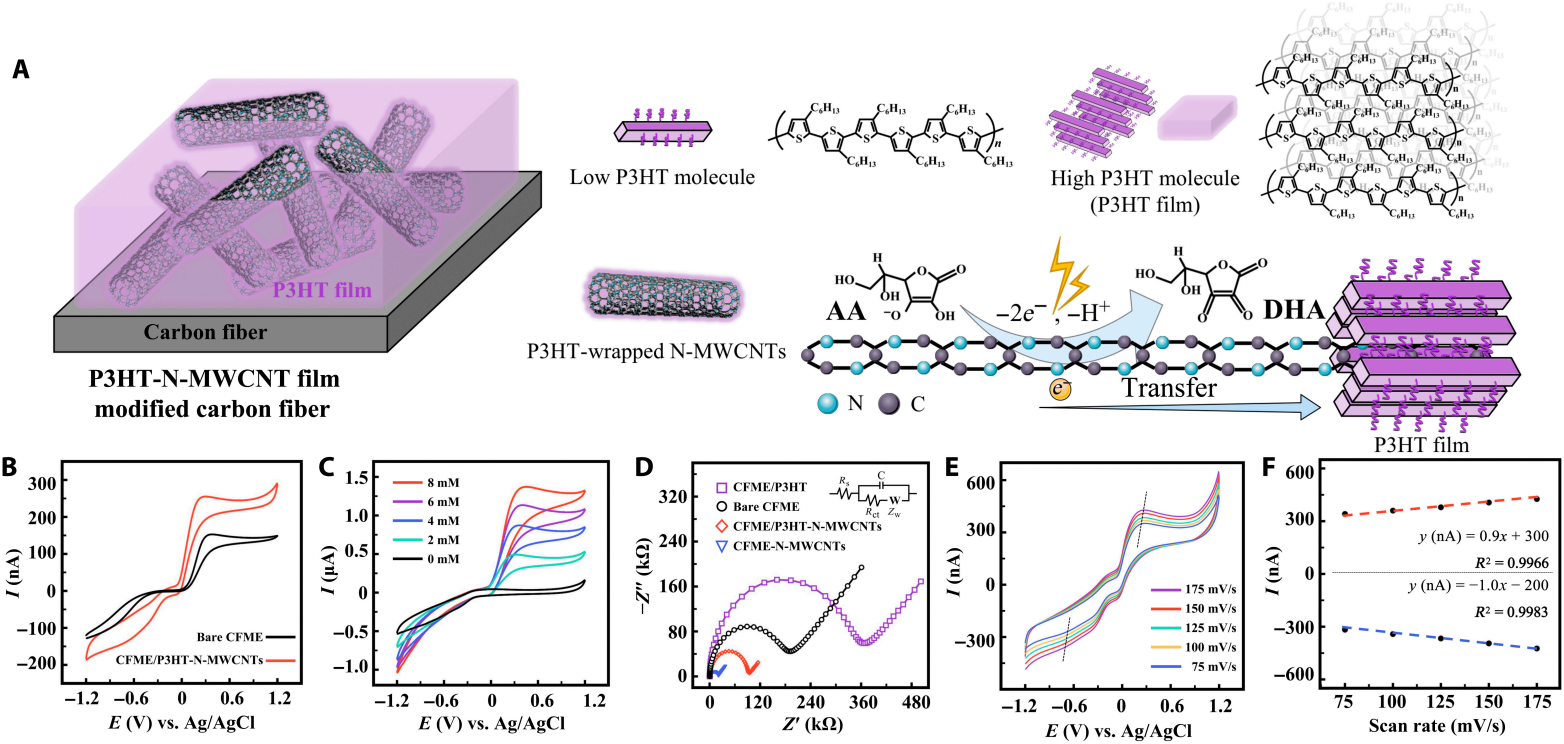
Electrocatalysis mechanism and electrochemical behavior of CFME/P3HT-N-MWCNTs. (A) Schematic description of the micromorphology of CFME/P3HT-N-MWCNTs. Top right scheme clarifies the structure of P3HT. Bottom right scheme clarifies the mechanism of AA-electrochemical oxidation and transfer of electrons at N-MWCNT surface. (B) CVs of AA (2 mM) at bare CFME and CFME/P3HT-N-MWCNTs. (C) CVs of different concentrations of AA at CFME/P3HT-N-MWCNTs. (D) EIS spectra of CFMEs measured in 0.1 M KCl containing 0.01 mM [Fe(CN)_6_]^3−/4−^. Inset is a Randles circuit for fitting. (E) CVs of the CFME/P3HT-N-MWCNTs at different scan rates. (F) The corresponding plot of current versus scan rate.

Using a one-pot technique, CFMEs are wrapped with a P3HT and N-MWCNT composite film (Fig. 1A and Fig. S1). Under Fe^3+^ oxidation, 3HT monomers at room temperature polymerize to form low P3HT molecules on the surface of CF and N-MWCNTs [40]. Further, part of the P3HT molecule, with more regular structures, can be generated by self-assembly to a high P3HT molecule (top right of Fig. 2A) [41]. Finally, low P3HT molecules, high P3HT molecules, and N-MWCNTs are stacked and wrapped with each other to form a thin, smooth, and regular membrane on CF (Fig. 1B). Figure S2 shows the energy-dispersive x-ray spectroscopy (EDS) spectrum of the composite film modified on CF, and the element distribution shows the modification of N-MWCNTs and the uniform encapsulation of the P3HT film.

For the 3HT polymerized on N-MWCNTs. Images of transmission electron microscopy (TEM) show that the N-MWCNTs wrapped in P3HT have a deeper hue and a less distinct edge (Fig. 1C and D). A high-angle annular dark-field scanning TEM (STEM) image and the corresponding elemental mapping analysis reveal the uniform distribution of C, N, O, and S elements. This phenomenon indicates the uniform distribution of N-MWCNTs on the CFME (Fig. 1E).

Thereafter, the chemical composition of CFME/P3HT-N-MWCNTs was analyzed by an x-ray photoelectron spectroscopy (XPS) full spectrum (Fig. 1F to J). The C 1s spectrum mostly contains 3 types of carbon atoms: C=C bond (sp2, 284.8 eV), C–C bond (sp3, 286.3 eV), and C–N bond (288.5 eV) (Fig. 1G). This finding reflects the composition of carbon element in electrode and the N-element doping on N-MWCNTs. As a commonly used modification method, nitrogen doping can improve the catalytic efficiency [42]; Fig. 1H shows the XPS spectral lines of electrode N elements after decomposition. Four structures of nitrogen atoms, namely, pyridine nitrogen (397.7 eV), pyrrolic nitrogen (399.1 eV), triply coordinated nitrogen (400.3 eV), and graphitic nitrogen (401.5 eV), are observed in the spectra of the electrode. Therefore, in addition to the nitrogen elements already present in CF and N-MWCNTs, stable nitrogen elements with triply coordinated structures are produced by N-MWCNT doping. The most substantial alloying impact on the sp2 carbon structure is exerted by the replaced nitrogen atom, which may effectively lower the impedance and accelerate electron transmission [43]. Furthermore, the material has 2 different structures of oxygen elements, C=O (531.7 eV) and C–OH (533.6 eV), which is related to the substance’s hydrophilicity (Fig. 1I) [44]. S species show a doublet structure in the S 2p core level spectrum, corresponding to the S 2p_1/2_ (164.7eV) and S 2p_3/2_ (163.9 eV) states that originate from the final state effect of spin multiplicity (Fig. 1J) [45]. These characterizations imply that N-MWCNTs and P3HT thin films are comodified on CF substrates.

### Electrocatalysis mechanism and electrochemical behavior of CFME/P3HT-N-MWCNTs

We also investigated the electrochemical performance of CFME/P3HT-N-MWCNTs to AA. P3HT and N-MWCNTs were modified on 200-μm CFMEs, which were exposed to NaOH. Figure 2B and Fig. S3 present the electrochemical oxidation of AA and the shape of electrode. Compared with bare CFME, the electrochemical oxidation of CFME/P3HT-N-MWCNTs to AA is improved marked. The oxidation currents range between 155 and 260 nA from peak to peak. The peak positions are advanced at +0.4 and +0.2 V, respectively. N-MWCNTs have outstanding electrochemical performance, which may be attributed to their high specific area, low overall electron impedance, and improved material transport and electrode electron transfer (bottom right of Fig. 2A) [46]. In addition, the P3HT film can accelerate the migration of charges produced via AA oxidation on carbon nanotubes and help transport electrons produced by N-MWCNTs to CF [47]. Subsequently, the performance of 2 mM AA in electrochemical oxidation at a glassy carbon electrode with or without composite film modifications is demonstrated. Further, a consistent trend with CFME is observed (Fig. S4), and the stability and repeatability of the modification strategy are proven.

CFME/P3HT-N-MWCNTs also exhibit a well-proportioned and persistent response to an increase in AA concentration, which is much higher than the physiology of rat brains (200 ± 50 μM) (Fig. 2C) [48,49]. This finding reflects the sensitivity and stability of the electrodes. The electrochemical impedance spectroscopy (EIS) results are presented in Fig. 2D, measured in the system of 0.1 M KCl solution containing 0.01 mM [Fe(CN)_6_]^3−/4−^. The P3HT film alone raises impedance levels as compared with bare CFME. However, composite film modification greatly lowers the impedance. Moreover, the examination of the N-element XPS spectra is supported by a obvious reduction in electron impedance caused by the alteration of N-MWCNTs alone.

Figure 2E displays the cyclic voltammograms (CVs) of CFME/P3HT-N-MWCNTs containing 2 mM AA in a cerebrospinal fluid (CSF) at different scan rates. As the scan rate increases, the anodic peak shifts slightly positively, but the cathodic peak shifts negatively. This finding indicates the rapid and reversible redox reaction of AA on the surface of CFME/P3HT-N-MWCNTs. Moreover, a tiny positive shift in the anodic spike potential suggests the tendency of the AA oxidation reaction to have a kinetic limit. Both the anodic and cathodic peak currents exhibit a linear relationship with an increasing scan rate from 75 to 175 mV/s. This situation proves the surface-adsorption-controlled process of mass transfer and interface load transfer between CFME/P3HT-N-MWCNTs and AA (Fig. 2F). Evidently, the ability of the composite membrane to transmit electrons is also improved, indicating favorable kinetics for the electrooxidation of AA at CFME/P3HT-N-MWCNTs. Overall, these findings demonstrate the electrochemical oxidation process of AA on the electrode (Fig. 2A).

### Evaluation of in vivo monitoring performance of CFME/P3HT-N-MWCNTs

**Fig. 3.**
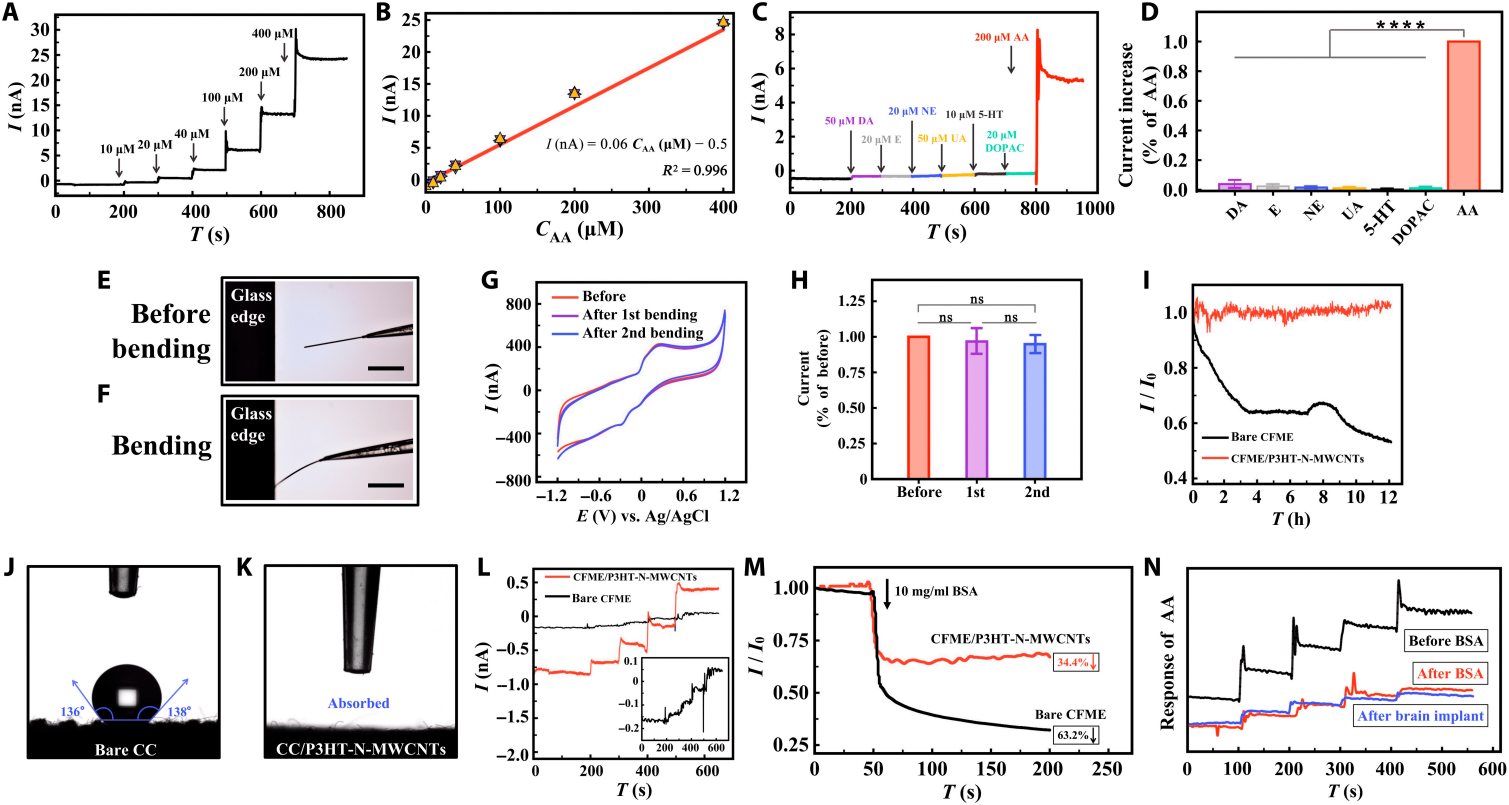
Evaluation of in vivo monitoring performance of CFME/P3HT-N-MWCNTs. (A) Amperometric response of CFME/P3HT-N-MWCNTs in aCSF to the successive addition of AA from 10 to 400 μM at +0.01 V versus Ag/AgCl and (B) the corresponding calibration curves (*n* = 5). (C) Selectivity of CFME/P3HT-N-MWCNTs toward AA at +0.01 V versus Ag/AgCl and (D) the corresponding statistical bar chart (*n* = 5). (E) and (F) are optical microscope images of CFME/P3HT-N-MWCNTs before bending and bending. Scale bars, 100 μm. (G) CVs of 2 mM AA in aCSF at CFME/P3HT-N-MWCNTs after each bending/recovering cycle (*n* = 3). (H) The normalized limiting oxidation current intensities of 2 mM AA in aCSF at CFME/P3HT-N-MWCNTs after each bending/recovering cycle (*n* = 3). (I) Amperometric response of 500 μM AA for 12 h recorded with bare CFME at +0.3 V and CFME/P3HT-N-MWCNTs at +0.01 V versus Ag/AgCl (*n* = 3). The contact angle measurements of bare CC (J) and CC/P3HT-N-MWCNTs (K) (*n* = 3). (L) Amperometric response of CFME/P3HT-N-MWCNTs and bare CFME in abandoned culture medium to the successive addition of AA at +0.01 and +0.3 V versus Ag/AgCl. Inset: Enlarged version of bare CFME (*n* = 3). (M) Amperometric response of CFME/P3HT-N-MWCNTs and bare CFME toward 200 μM AA in aCSF with BSA (10 mg/ml) addition at +0.01 and +0.3 V versus Ag/AgCl (*n* = 3). (N) Amperometric responses of CFME/P3HT-N-MWCNTs toward successive addition of AA before BSA treatment (10 mg/ml), after BSA treatment (10 mg/ml), and after brain implant for 2 h in aCSF (*n* = 3) (ns, nonsignificant; ****P < 0.0001). DA, dopamine; NE, norepinephrine; DOPAC, 3,4-dihydroxyphenylacetic acid; UA, uric acid; E, epinephrine; 5-HT, 5-hydroxytryptamine.

We evaluated whether CFME/P3HT-N-MWCNTs maintain a specific and stable response to AA in vivo. First, differential pulse voltammetry was used to examine the oxidation of electroactive substances that commonly coexist in the brain (Fig. S5). The detected species and their oxidation initiation potentials are AA (−0.09 V), dopamine (0 V), norepinephrine (0.028 V), 3,4-dihydroxyphenylacetic acid (0.01 V), uric acid (0.2 V), epinephrine (0 V), and 5-hydroxytryptamine (0.21 V) (versus Ag/AgCl) in this order. Evidently, the oxidation initial potentials of most interferences are close to 0.01 V (versus Ag/AgCl). Because of the clear advancement of its oxidation potential, AA exhibited a considerable current response at 0.01 V (versus Ag/AgCl). Meanwhile, given the intricacy of the chemical composition used in living brains, the signaling potential for further investigations displays a higher selectivity of 0.01 V (versus Ag/AgCl) in the physiological environment of the brain. Figure 3A and B shows the detection linearity of CFME/P3HT-N-MWCNTs, covering the AA concentration in physiological environments and having a range of 10 to 400 μM (*I*/nA = 0.06*C*_AA_/μM − 0.5, *R*^2^ = 0.996) [50].As demonstrated, the electrode responds to AA with sensitivity and accuracy. Simultaneously, CFME/P3HT-N-MWCNTs have high selectivity for AA following the detection potential adjustment (Fig. 3C and D). Therefore, the oxidation signal of other neuroactive molecules in the electrode can be ignored. Nearly all significant electrical signals can be considered AA responses.

Subsequently, the stability of CFME/P3HT-N-MWCNTs was assessed. The peak potential at 0.20 V of CVs was recorded after each bending of CFME/P3HT-N-MWCNTs against the edge of a glass slide to examine the mechanical resistance of the electrode. Figure 3E and F shows the images of an electrode tip obtained from an optical microscope. The shape of the implanted components is also shown, and the deformation performance of CFME/P3HT-N-MWCNTs is established. The CV curves are observed to nearly coincide before and after each bending (Fig. 3G). Simultaneously, the change in peak oxidation current is insignificant, and the current is approximately 96.7% ± 6.8% and 94.9% ± 2.9% after bending once and twice, respectively (Fig. 3H). Therefore, CFME/P3HT-N-MWCNTs have good flexibility and rigidity, and the electrode cannot be severely damaged by force. Thereafter, the capacity for continuous monitoring of CFME/P3HT-N-MWCNTs was evaluated over the long term in vitro (Fig. 3I). The bare CFME presents a full downtrend, with a 47% decrease in the amperage response. However, CFME/P3HT-N-MWCNTs can respond steadily for 12 h, and the overall current is almost identical to the initial current, proving the durable, stable, and sensitive response of the electrode to AA (500 μM) in the rat CSF. Thus, CFME/P3HT-N-MWCNTs exhibit excellent stability, mechanical force tolerance, and dependability and establish a strong basis for the ongoing monitoring of living tissues.

Antifouling is one of the greatest challenges for implantable electrodes because immune cells and nonspecific proteins can occupy active electrode locations, causing a constant loss of sensitivity. P3HT-N-MWCNTs have 2 different structures of oxygen structures, namely, C=O (531.7 eV) and C–OH (533.6 eV). This configuration provides the material with good hydrophilicity, helping to improve the fouling resistance of the electrode (Fig. 1I). We investigated the antifouling performance of CFME/P3HT-N-MWCNTs. We first measured the water contact angles of carbon cloths (CC) modified with and without P3HT-N-MWCNT composite films to assess the hydrophilicity (Fig. 3J and K). The water droplets eventually formed 136° and 138° angles and were stable and difficult to attach to bare CC surfaces. However, CC/P3HT-N-MWCNTs were rapidly and completely absorbed when they were touched by droplets on the surface. Two videos without speed adjustment are provided to visualize the difference between them (Movies S1 and S2). The hydrophilicity of the electrode can be considerably improved by modifying the P3HT-N-MWCNT composite film. Consequently, the protein on the electrode surface has a higher affinity for water, and the electrode is less likely to become polluted. The entire medium collected from HT22 cells for 2 days was used to soak CFME/P3HT-N-MWCNTs and bare CFME for 30 min. Thereafter, the responses of electrodes to 10 to 100 μM AA were compared (Fig. 3L). Greater AA sensitivity is observed for bare CFME at 0.3 V than for CFME/P3HT-N-MWCNTs at 0.01 V potential (Fig. S3A and D). Figure 3L (black curve) shows the result of the evidently low sensitivity and instability. The possible reasons are the following: (a) the low AA selectivity of bare CFME without detection potential optimization (hence, the active sites are easily to be occupied by other substances in the solution environment) and (b) electrode contamination by various cellular metabolic wastes, exosomes, and serum proteins in the discarded medium. However, CFME/P3HT-N-MWCNTs still show a stable and specific response to AA. To further evaluate the kinetics at instant of electrode contamination, we added bovine serum albumin (BSA; 10 mg/ml) to a CSF containing 200 μM AA (Fig. 3M). In particular, the bare CFME is inherently unstable both before and after the addition of BSA, and the response signal is decreased by approximately 63.2%. Meanwhile, CFME/P3HT-N-MWCNTs always maintain a smooth electrochemical signal to AA, and the current value is only reduced by 34.4%, proving the certain fouling resistance of the CFME modified with a composite film. Then, BSA (10 mg/ml) was used to pretreat CFME/P3HT-N-MWCNTs to bind parts of the active site to keep the in vivo detection stable [51]. The amperometric responses of the electrode before BSA pretreatment, after BSA pretreatment, and 2 h after brain implantation were compared (Fig. 3N). When implanted in brain tissue for 2 h after BSA treatment, CFME/P3HT-N-MWCNTs maintain their initial reaction to AA, and their sensitivity does not drastically decrease. Considering the findings presented in Fig. 3M, we believe that this result is the consequence of the one-pot modification approach. This is because some N-MWCNTs are present on the electrode surface but are not entirely covered by the P3HT layer. Further, pretreatment can bind this part of the active sites and stabilize the electrode. Therefore, altering the P3HT-N-MWCNT composite film may increase the CFME’s sensitivity and stability in a physiological setting. In addition, the composite film has some antifouling features. An example of a real-world application of the antifouling properties of P3HT film is brain implantation following pretreatment with CFME/P3HT-N-MWCNTs.

### Real-time monitoring of AA release from nerve cells and brain slices in a through-flow groove

**Fig. 4.**
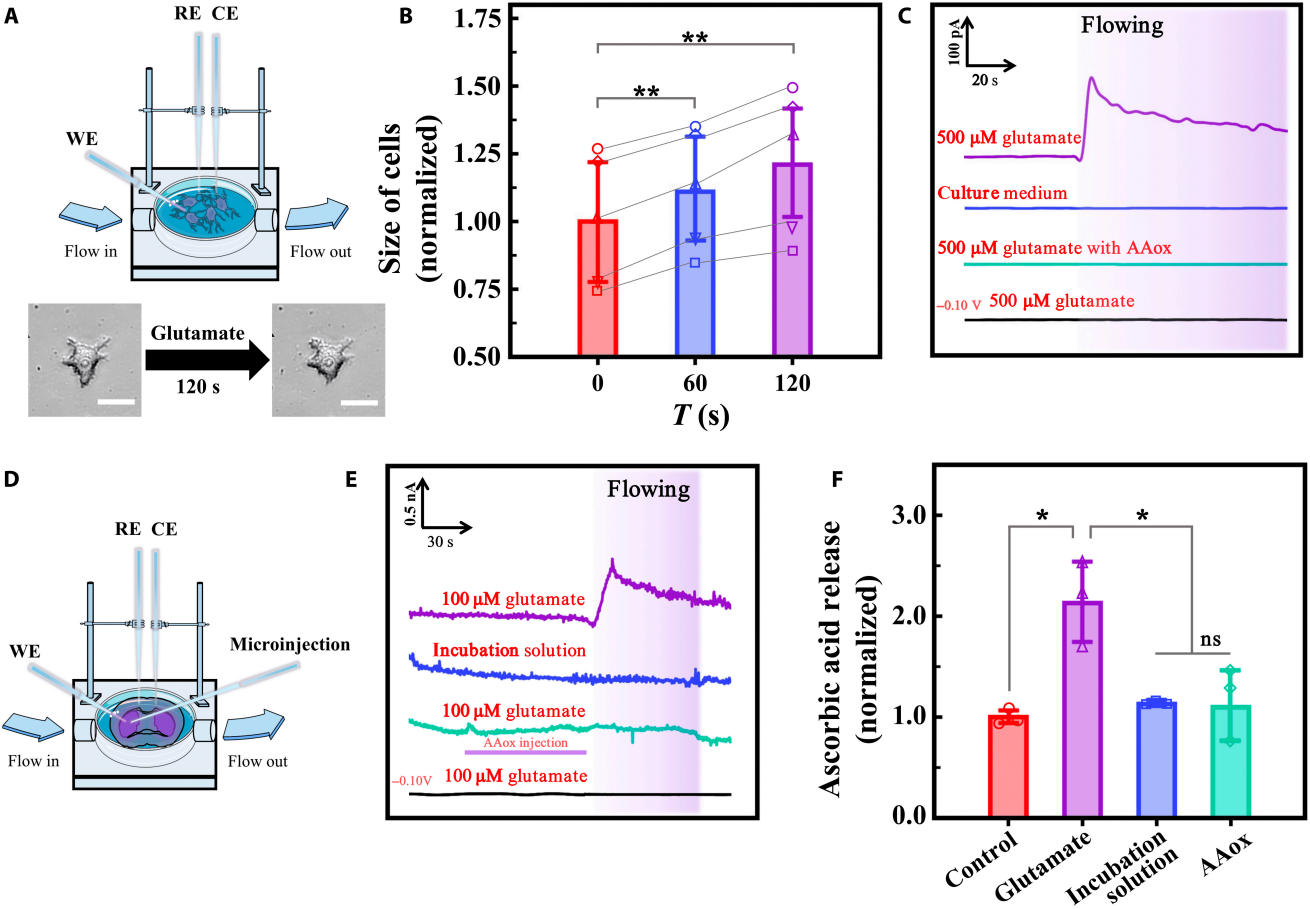
Real-time monitoring of AA release from nerve cells and brain slices in a through-flow groove. (A) Schematic description of HT22 cells monitoring in through-flow groove. Bottom: Size changes of individual HT22 cell before and after 120-s glutamate flowing. Scale bars, 25 μm. RE, reference electrode; CE, counter electrode. (B) HT22 cells’ size treated by 500 μM glutamate at different times (*n* = 5, each for 5 cells). (C) Amperometric response of AA in HT22 cells with flowing infusion (0.5 ml/min for 150 s) of 500 μM glutamate (purple), culture medium (blue), 500 μM glutamate with AAox (green), and 500 μM glutamate at −0.10 V (black), from top to bottom. The flowing durations are indicated by purple background (*n* = 5). (D) Schematic description of brain slice monitoring in through-flow groove. (E) Amperometric response of AA in striatum of rat brain slices with CFME/P3HT-N-MWCNTs at 0.01 V alone flowing infusion (2 ml/min for 60 s) of 100 μM glutamate (purple), incubation solution (blue), 100 μM glutamate with AAox injection (1 μl/min for 60 s) (green), and 100 μM glutamate at −0.10 V (black), from top to bottom (*n* = 3). The flowing durations are indicated by purple background, and the injection duration is marked by lines. (F) The corresponding AA concentration statistics. *P < 0.05 and **P< 0.01.

Because of the excellent performance of CFME/P3HT-N-MWCNTs demonstrated in the aforementioned trials, we could analyze the release process of AA in the rat brain. First, a through-flow groove was constructed to simulate the changes under similar physiological conditions in the brain (Fig. 4A). It can also monitor the electrochemical signal by the working electrode (WE), reference electrode, and counter electrode. In the field of WE, microinjections can also be given simultaneously. Both HT22 cells and brain slices can be used to monitor AA dynamics release (Fig. S6).

Glutamate, a common excitatory amino acid in the neurological system, can lead to an increase in AA concentration in the intercellular fluid when its content in the brain increases [52–54]. First, the electrodes were inserted into HT22 cells immersed in complete Dulbecco’s modified Eagle medium (DMEM) (Fig. 4A). When 500 μM glutamate infusion was initiated, significant swelling occurred in HT22 cells (Fig. 4A and B). Second, a significant AA signal was detected by the electrode after the addition. The outcomes demonstrate that AA could be monitored using CFME/P3HT-N-MWCNTs in a real cellular setting when cytotoxic edemas occurs (Fig. 4C).

For AA measurement in the brain, the tip of CFME/P3HT-N-MWCNTs was fully implanted into the striatum of brain slices. Figure S7 illustrates the electrochemical response within 1 h, demonstrating the electrode’s durability and the excellent vitality of the brain slices. Then, 1 mM AA and incubation solution were poured into the through-flow groove (Fig. S8). The results show that AA gradually enters brain cells and builds up to its maximal dynamic process. The CFME/P3HT-N-MWCNT is also sensitive to AA in brain tissue.

Furthermore, 100 μM glutamate was injected by the microinjection pump from left side to right side to replicate the fluctuation in glutamate levels in the interstitial fluid of the brain due to diseases or physiological situations. Before the experiment, we proved that glutamate is insensitive to CFME/P3HT-N-MWCNTs (Fig. S9A). Figure 4E presents the AA signal obtained after 100 μM glutamate was injected. Brain slices were placed into the incubation solution before adding glutamate to discover the physiological concentration of AA in the brain slices via CFME/P3HT-N-MWCNTs. When glutamate was administered, the AA concentration rapidly increased to 2.13 ± 0.40 times the physiological concentration and then gradually decreased to a stable level. Within 1 mm of the brain tissue where the WE was implanted, ascorbate oxidase (AAox) was microinjected in an ultramicroinjetion pump to quickly oxidize the resultant AA. Only 1.10 ± 0.29 times more AA was present following glutamate addition. Furthermore, no AA signals can be observed in brain slices before or after glutamate injection at −0.10 V, wherein AA cannot be electrooxidized. When the brain slices were perfused with an incubation solution at +0.01 V, no significant AA release was observed. The concentration was only 1.13 ± 0.02 times that of the physiological state. A statistical comparison between the control and +0.01 V groups reveals a significant difference. Therefore, it can be fully proved that the significant electrochemical response produced after adding of 100 μM glutamate is an AA release. In general, the actual detection effect of the electrode on AA release from brain slices under physiological changes was obtained.

We further compared the biocompatibility of the materials to judge whether CFME/P3HT-N-MWCNTs is suitable for long-term implantation in vivo. Considering the oxidative stress caused by the residual Fe^3+^/Fe^2+^ [55], we observed the distribution of iron element in the electrode and proved its presence inapparent (Fig. S2E). Then, the composite membrane was modified on carbon cloths and implanted subcutaneously for 2 weeks. The response of the P3HT-N-MWCNT composite membrane to skin tissues was compared using an immunohistochemical method in coronal sections. We explored the existence of activated macrophage differentiation cluster 68 (CD68) and mannose receptor C-type 1 (MRC1), which represent activated macrophages and alternatively activated macrophages (M2 macrophages), respectively. The peripheral biomarker expression of CC/P3HT-N-MWCNTs, CC/N-MWCNTs, and CC/P3HT-linked subcutaneous tissues and the tissue responses are comparable to those observed for bare CC (Figs. S10 and S11). As demonstrated, the microelectrode has a modest reaction to foreign bodies and strong histocompatibility. Meanwhile, in the CCK-8 experiment, no significant difference exists in the growth of HeLa cells between bare CC and CC/P3HT-N-MWCNTs (Fig. S12). Calcein acetoxymethyl (Calcein AM) and propidium iodide (PI) fluorescent dyes were used to check living cells and dead cells (Fig. S13). Both bare CC and CC/P3HT-N-MWCNTs show abundant cell growth and negligible cells death. In addition, equal amounts of HeLa-red fluorescent protein (RFP) cells were also cocultured on bare CC and CC/P3HT-N-MWCNTs for 6 h. Cells can survive normally on both bare CC and CC/P3HT-N-MWCNTs, indicating that the cytotoxic of our material modification strategy is not obvious (Fig. S14).

### Investigating the possible mechanism of AA changes in brain intercellular fluid with glutamate-induced edemas

After discovering that the addition of glutamate triggers AA release and cell edema, we explored the relationship between the 2 phenomena. We used the NMDAR and Cl^−^ channel blockers MK-801 and 4,4-diisothiocyanatostilbene-2,2′-disulfonic acid (DIDS), respectively, to prevent the entry of Na^+^ and Cl^−^ core units that were responsible for this process. The aim was to explore the direct connection between this process and the occurrence of cytotoxic edemas. We also monitored the release of AA caused by glutamate excitation.

**Fig. 5.**
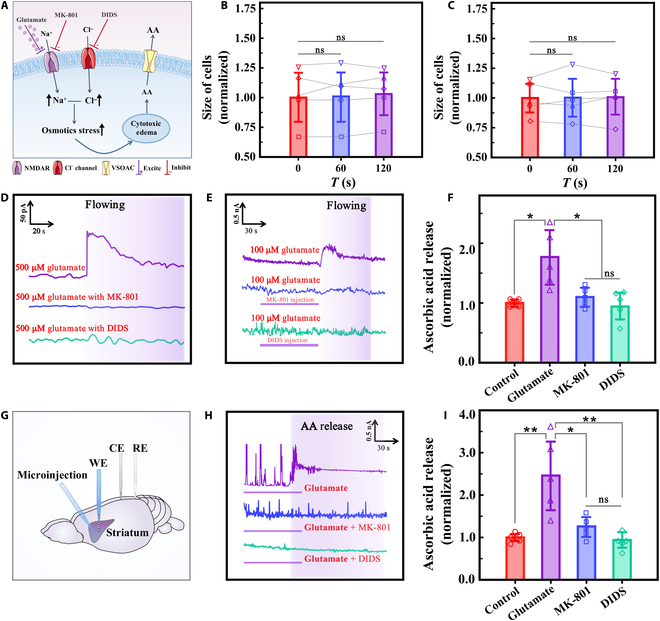
Investigating the possible mechanism of AA changes in brain intercellular fluid with glutamate-induced edemas. (A) Schematic illustration of the possible mechanism of AA release during cytotoxic edema. (B) HT22 cells’ size treated by 500 μM glutamate with 1 mM MK-801 at different times (each for 5 cells). (C) HT22 cells’ size treated by 500 μM glutamate with 5 mM DIDS at different times (each for 5 cells). (D) Amperometric response of AA to in vitro HT22 cells with CFME/P3HT-N-MWCNTs at 0.01 V alone flowing infusion (0.5 ml/min for 150 s) of 500 μM glutamate (purple), 500 μM glutamate with 1 mM MK-801 (blue), and 500 μM glutamate with 5 mM DIDS (green), from top to bottom. The flowing durations are indicated by purple background. (E) Amperometric response of AA in striatum of ex vivo rat brain slices with CFME/P3HT-N-MWCNTs at 0.01 V alone flowing infusion (2 ml/min for 60 s) of 100 μM glutamate (purple), 100 μM glutamate with 1 mM MK-801 microinjection (1 μl/min for 60 s) (blue), and 100 μM glutamate with 5 mM DIDS microinjection (green), from top to bottom. The flowing durations are indicated by purple background and the injection duration is marked by lines. (F) The corresponding AA concentration statistics. (G) Schematic description of in vivo monitoring. (H) Amperometric response of AA in striatum of in vivo monitoring with CFME/P3HT-N-MWCNTs at 0.01 V alone microinjection (1 μl/min for 60 s) of 100 μM glutamate (purple), 100 μM glutamate with 1 mM MK-801 microinjection (blue), and 100 μM glutamate with 5 mM DIDS microinjection (green), from top to bottom. The injection duration is marked by lines, and AA release is indicated by a purple background. (I) The corresponding AA concentration statistics (*n* = 5). *P < 0.05 and **P < 0.01.

Here, we first investigated the process of glutamate-induced cytotoxic edemas at the cellular level. Before the formal experiment, we had previously demonstrated that MK-801 and DIDS will not be responded by the electrode (Fig. S9B and C). By successively adding 500 μM glutamate, 500 μM glutamate with 1 mM MK-801, and 500 μM glutamate with 5 mM DIDS, we observed the degree of edema occurrence in 5 cells from 5 dishes (Fig. S15). Figures 4B and 5B and C show the cells’ size changes. Significant swelling was observed in the cells treated with glutamate alone but not in the MK-801 and DIDS groups. Thus, Na^+^ and Cl^−^ influx was possibly inhibited, resulting in limited cell swelling. Simultaneously, we monitored the AA release process during the treatment of the cells with the 3 groups of drugs. Evidently, the cells treated with MK-801 and DIDS barely released AA (Fig. 5D). Therefore, we believe that AA release is directly related to the cytotoxic edema process caused by glutamate. Thereafter, we replicated the experiments on brain slices to explore the plausibility of this mechanism.

We placed the CFME/P3HT-N-MWCNTs implants in the through-flow groove of the striatum of the rat brain slices to constantly keep track of how much AA is changing in the tissues. A microinjection needle was inserted 1 mm distant from the WE to add the inhibitors. The untreated group had an immediate response in terms of AA release, which is consistent with that in Fig. 4E, following glutamate infusion. Therefore, the significant AA concentration generated by NMDAR excitation increased to a peak and then stabilized (Fig. 5E and F) with AA concentration, which was approximately 1.77 ± 0.47 times that of the physiological concentration. The excitatory effect of glutamate on NMDAR can be antagonized by MK-801, and the AA efflux was reduced to only 1.10 ± 0.16 times the physiological concentration when MK-801 was preinjected. We hypothesize that glutamate is responsible for the inhibition of NMDAR-mediated Na^+^ influx, the reduction in osmotic pressure elevation, and the blockade of membrane potential depolarization in neurons. Further, the cytotoxic edema process was inhibited, and the activation of VSOACs was reduced, preventing a considerable increase in the AA signal in the intercellular fluid. How inhibiting NMDAR and cytotoxic edema processes directly affects AA release was also demonstrated. DIDS can effectively inhibit cytotoxic edemas by blocking HCO_3_^−^/Cl^−^ exchange. By blocking the Cl^−^ channel and reducing the Cl^−^ influx, glutamate’s ability to generate intracellular osmotic pressure can be minimized. Therefore, AA release was greatly inhibited by 0.94 ± 0.23 times of the physiological concentration. Our theory on the mechanism of glutamate-induced AA release is supported by the ability to detect AA release and efficiently suppress cytotoxic edemas when Cl^−^ channels are blocked.

Finally, we used a cranial drill to expose part of the brain after anesthetizing the rat. The counter electrode and reference electrode were implanted, CFME/P3HT-N-MWCNTs were implanted into the striatum as the WE, and the microinjection pump was implanted 1 mm away from the WE (Fig. 5G). After microinjection of 100 μM glutamate, the electrode produced a sustained and significant current response. Its current intensity was approximately 2.46 ± 0.83 times the physiological AA concentration and significantly different from that of the control group (Fig. 5H and I). Next, MK-801 was added to study the electrochemical behavior of the in vivo release of AA after NMDAR blockade. The results were only manifested as tiny physiological releases of AA, and the current was only 1.26 ± 0.25 times that of the physiological concentration. We used DIDS to block Cl^−^ influx to completely inhibit edema occurrence. Only physiological fluctuation of AA was detected, and the current was 0.94 ± 0.18 times the physiological concentration. No significant differences are found between microinjection of 100 μM glutamate solution containing 1 mM MK-801 and 5 mM DIDS, but both of them differ significantly from that of glutamate alone. This finding is consistent with our results in HT22 cell detection and brain slice monitoring.

Therefore, with stimulation and blocking, NMDAR can serve as a key receptor to effectively control glutamate-induced release of AA from neuronal cells via cytotoxic edema mechanisms. By limiting the Na^+^ and Cl^−^ influx, the increase in the intracellular osmotic pressure and the process of cytotoxic edemas of the nerve cells are blocked (Fig. 5A). The final manifestation is diminished by AA release. The conclusions presented above are strongly supported by real-time electrochemical in situ monitoring of glutamate-induced AA release in vitro in HT22 cells, ex vivo in brain slices, and in vivo in living rats. Overall, we have proven that glutamate induces AA release via NMDAR activation and that this process is controlled by the cytotoxic edema process of Na^+^ and Cl^−^ influx induced by glutamate. These results provide new evidence for the mechanism of early pathological changes in cerebral edema, neuroexcitotoxicity of glutamate, and dynamics of AA-induced cytotoxic edema.

## Discussion

The in situ electrochemical monitoring of biological tissues needs to achieve linear detection over physiological or even pathological concentrations of markers while maintaining stability [56,57]. As AA is easily oxidized, it must be monitored in real time at the release site to ensure accuracy, and the implementation of this technology has high requirements for electrode performance. Typical issues include insufficient electrode specificity and the active site’s susceptibility to nonspecific protein adsorption, which continuously lowers sensitivity and stability. Numerous excellent membrane materials have been utilized to improve sensor performance, and we have sufficiently compared all membrane-modified biosensors applied to AA monitoring (Table S1). In this study, we comprehensively evaluated the adaptability of P3HT for electrochemical sensing and confirmed its overall superior performance. By using a one-pot approach at room temperature and atmospheric pressure, we fabricated a CFME/P3HT-N-MWCNT microelectrode for AA in vitro and in vivo monitoring. The microelectrodes have a favorable morphology, a high catalytic performance for AA, and a large capacity for charge transport. Hence, they exhibit outstanding linearity, selectivity, stability, stain resistance, and biocompatibility. In addition, the electrodes are able to specifically respond linearly to ascorbate while remaining stable against protein adsorption in brain brimming with complicated chemicals.

Then, CFME/P3HT-N-MWCNTs were further applied to monitor AA in HT22 cells and brain slices in a through-flow groove platform and demonstrate excellent detection performance for monitoring neurochemicals in vivo. Glutamate is a common excitatory amino acid in the nervous system; when its concentration exceeds the physiological level, it causes excitotoxicity and nerve damage [30]. The release of AA occurs when glutamate is injected into the brain. The 2 specific mechanisms involved are the heterogeneous exchange between glutamate and AA [38,39] and brain edema, a hypothesis that has been proposed in recent years [34]. In particular, researchers have hypothesized that glutamate could activate VSOAC by inducing cell swelling, resulting in the release of AA [29,34,35]. However, no researchers have directly investigated the relationship between the edema-inducing effect of glutamate and the release of AA, and much less has the mechanism been revealed. By controlling the switching of NMDAR and Cl^−^ channels, we monitored AA release in glutamate-induced cerebral edema and discovered that glutamate-induced cerebral edema and AA were released by activating NMDAR and increasing Na+ and Cl− inflow to exacerbate osmotic stress. This situation increases the release of AA by causing cytotoxic edemas. This study is the first direct observation and demonstration of the mechanism of cerebral edema underlying glutamate-induced AA release. Therefore, with a composite membrane modification of a neuroelectrochemical sensing technique, AA and other neurochemicals in complex physiological conditions may be tracked. They have broad applications potential for electrochemical sensing of animals or cells, which is helpful in elucidating incomprehensible neuroscientific mechanisms. As the advancement and development of bioelectrochemical in situ sensing technology, this study can serve as a reference and a source of inspiration for biosensing and elevate the value and standing of this technology in the life sciences.

## Materials and Methods

### Reagents and solutions

N-MWCNTs was purchased from XFNANO Materials Tech Co. Ltd. (Jiangsu, China). 3HT was bought from TCI (Shanghai, China). Iron(III) chloride hexahydrate was obtained from Shanghai Macklin Biochemical Co. Ltd. (Shanghai, China). Chloroform, methyl alcohol, ethanol, acetone, nitric acid, and potassium hydroxide were purchased from Chongqing Chuandong Chemical Group Co. Ltd. (Chongqing, China). AA, adrenaline, norepinephrine, 3,4-dihydroxyphenylacetic acid, dopamine, 5-hydroxytryptamine, uric acid, AAox, l-glutamate, MK-801, and DIDS were obtained from Sigma-Aldrich (St. Louis, MO, USA), and were prepared in ultrapure water (18.2 MΩ). Artificial CSF (aCSF) was prepared by dissolving NaCl (126 mM), KCl (2.4 mM), KH_2_PO_4_ (0.5 mM), MgCl_2_ (0.85 mM), NaHCO_3_ (27.5 mM), Na_2_SO_4_ (0.5 mM), and CaCl_2_ (1.1 mM) in ultrapure water, and the solution was adjusted to pH 7.4. CD68 and 4′,6-diamidino-2-phenylindole were bought from Beyotime (Shanghai, China). CD206 (i.e. MRC1) was bought from Proteintech (Hubei, China).

### Apparatus and measurements

Unless otherwise noted, all studies were conducted at room temperature. The electrochemical potential measurements were conducted using a computer-controlled CHI 630e electrochemical analyzer (Chenhua Instruments Co., Shanghai, China). CFMEs and glassy carbon electrode, Pt wire, and Ag/AgCl (3 M KCl) electrode were used as WEs, counter electrode, and reference electrode, respectively, together which formed a 3-electrode system for electrochemical experiments. Morphological characterizations of WEs were performed using a scanning electron microscope (SEM) on a SU8010 (Hitachi, Tokyo, Japan). A static water contact angle measuring analyzer Theta Flex (Biolin, Gothenburg, Sweden) was used to measure the materials’ hydrophilic characteristics. To evaluate the preparation materials, an XPS K-Alpha (Thermo Fisher Scientific, Waltham, USA) analysis was performed.

### CFME preparation

CFMEs (7 μm in diameter) were fabricated as described. Briefly, a glass capillary (inside diameter, 1.5 mm; length, 100 mm) was pulled from the using a micropipette puller (MP-500, RWD, Shenzhen, China) to created 2 tapered glass-coated CF tip electrodes, and the fine tip of each was broken into 10 to 15 μm in diameter. The pulled capillary was used as the sheath of CFMEs. Then, the CF (7 μm in diameter; Shenzhen Tony Electronics Co. Ltd., Shenzhen, China) was sonicated in acetone, 3 M HNO_3_, 1 M KOH, and ultrapure water (18.2 MΩ) sequentially, each for 5 min. In addition, they were dried in a vacuum-drying oven at 60 °C for 6 h. A single CF on a clean glass plate was attached to a copper wire (200 μm in diameter) slightly longer than the glass capillary with silver conducting paste and placed it at 80 °C for 1 h. Then, CFMEs were made by carefully inserting the CF attached copper wire into the capillary with CF exposed to the fine open end of the capillary and Cu wire exposed to the other end of the capillary. Next, immerse the end of the glass capillaries vertically in the molten paraffin pool, taking care that the paraffin does not touch the CF at the tip and then places it in room-temperature air to solidify the paraffin. Finally, the exposed CFMEs were cut to ca. 200 to 250 μm with a surgery scalpel under a microscope.

### Fabrication of CFME/P3HT-N-MWCNTs sensor

CFME/P3HT-N-MWCNTs sensors were prepared by in situ polymerization. Before the formal modification of the electrode surface, a mixed solution of 3HT and N-MWCNTs should be prepared initially. The preparation of the mixed solution mainly included the following steps. In one volume of chloroform (0.9 ml), N-MWCNTs (0.038 g, 2.8 wt%) and 3HT (45 μl, 28 μM) were added. Excess iron(III) chloride hexahydrate was then added to 2 volumes of chloroform separately (1.8 ml), and Fe^3+^ in the solution promoted the production of 3HT polymers on N-MWCNTs and electrode surfaces. After that, the 2 solutions were then respectively sonicated for 1 h each to facilitate dispersion and dissolution. After ultrasonication, the saturated ferric chloride solution was filtered out and placed into the N-MWCNTs and 3HT mixed solution, which was agitated equally.

Before modification, CFMEs were electrochemically activated to make their surfaces activate, firstly by potential-controlled amperometry in 1 M NaOH at +1.5 V for 80 s and then by cyclic voltammetry with a potential range from −1.2 to 1.2 V at a scan rate of 50 mV/s until a stable CV was obtained. The CFME was immersed in a mixed solution of 3HT, N-MWCNTs, and iron(III) chloride hexahydrate for 72 h. Finally, we washed the CFME with ultrapure water (18.2 MΩ) until the washing solution is colorless and the CFME is dry at room temperature.

The as-prepared electrode is denoted as CFME/P3HT-N-MWCNT electrode.

### In vitro HT22 cells experiments

All of the HT22 cells were cultured in DMEM with 1% penicillin-streptomycin solution, 500 μM AA, and 10% fetal bovine serum at 37 °C in 5% CO_2_. First, we implanted the 3-electrode system into the complete medium containing cells. At the beginning of the experiment, we perfused DMEM containing 500 μM glutamate, 500 μM glutamate with 1 mM MK-801, and 500 μM glutamate with 5 mM DIDS. The obtained current response was recorded.

When investigating the factors influencing cells edema, medium containing 500 μM glutamate, 500 μM glutamate with 1 mM MK-801, and 500 μM glutamate with 5 mM DIDS were added to separate HT22 cells dishes, respectively. Recorded cells images at 0, 60, and 120 s after adding the solution. ImageJ was used to count the size of the 5 cells in each image separately and calculate the change.

### Ex vivo brain slices experiments

The animal experiments were performed in accordance with the Ethic Committee of Chongqing Medical University. Before decapitation, adult Sprague-Dawley rats (male, 300 to 350 g) were anesthetized using isoflurane (4% for induction and 2% for maintenance) through a gas pump R520 (RWD Life Science Co. Ltd., Shenzhen, China), brains were rapidly extracted (<60 s) in 0 to 5 °C ice-cold slicing solution containing : NaCl (119 mM), KCl (2.5 mM), NaH_2_PO_4_ (1.25 mM), NaHCO_3_ (26 mM), glucose (10 mM), MgCl_2_ (7 mM), CaCl_2_ (0.5 mM), and AA (1.0 mM), pH 7.0; saturated with 95% O_2_/5% CO_2_. After trimming the brain tissue and fixing it on the pedestal of the slicer, coronal corticostriatal slices, 500 μm, were sliced using a vibrating tissue slicer (KD-400, Zhejiang Jinhua Kedi Instrumental Equipment Co. Ltd., Zhejiang, China). Four or 5 slices were collected and incubated at 34.5 °C in incubation solution containing: NaCl (119 mM), KCl (2.5 mM), NaH_2_PO_4_ (1.25 mM), NaHCO_3_ (26 mM), glucose (10 mM), MgCl_2_ (2.0 mM), CaCl_2_ (2.0 mM), and AA (1.0 mM), pH 7.4; saturated with 95% O_2_/5% CO_2_ for 30 min to restore cellular states.

The incubated brain slices were then transferred to a through-flow groove, along with 2 ml of incubation fluid. To hold the WE and puncture the coronal corticostriatal slice, a 3-axis electric micromanipulator MM-500 (RWD Life Science Co. Ltd., Shenzhen, China) was used. The microsized Ag/AgCl (KCl-saturated) reference electrode and Pt wire were positioned below the liquid level. To ensure the constant renewal of the solution system, 2 microsyringe pumps were utilized to inject the test solution and withdraw the perfusion tank solution at the same rate (2 ml/min) on the left and right, respectively, when the perfusion experiment began. In addition, a glass microelectrode injection pump R480 (RWD Life Science Co. Ltd., Shenzhen, China) was used to inject drugs locally. Before use, all test solutions were saturated with 95% O_2_/5% CO_2_ and heated to 34.5 °C.

### In vivo living rat brain experiments

The animal experiments were performed in accordance with the Ethic Committee of Chongqing Medical University. Before experiment, adult Sprague-Dawley rats (male, 300 to 350 g) were anesthetized using isoflurane (4% for induction and 2% for maintenance) through a gas pump R520 (RWD Life Science Co. Ltd., Shenzhen, China). The rat was immobilized using a brain stereotaxic apparatus 68000 (RWD Life Science Co. Ltd., Shenzhen, China), and the skin was cut to expose the skull after shaving the fur on the top of the head with the aid of a digital microscope DOM-1001 (RWD Life Science Co. Ltd., Shenzhen, China) and leveling. The body temperature was then maintained at 37 °C using a thermostatic blanket. We first used cranial drills 78001 (RWD Life Science Co. Ltd., Shenzhen, China) to drill holes in the skull and then used 3-axis electric micromanipulator MM-500 (RWD Life Science Co. Ltd., Shenzhen, China) to insert counter and reference electrodes into the cortex. CFME/P3HT-N-MWCNTs was implanted in the striatum (AP = 0 mm, L = 3.0 mm from bregma, V = 4.5 mm from Dura), and needle of glass microelectrode injection pump R480 (RWD Life Science Co. Ltd., Shenzhen, China) was implanted within 1 mm horizontally AP, L, and V represent anteroposterior, mediolateral, and dorsoventral, respectively.

Glutamate (100 μM), glutamate (100 μM) + MK-801 (1 mM), and glutamate (100 μM) + DIDS (5 mM) were injected near the electrodes after the experiment began, respectively. The CHI 630e (Chenhua Instruments Co., Shanghai, China) was used to monitor the current of AA.

### Subcutaneous implantation experiment and histological analysis

The experimental protocol and operation were approved by the Ethic Committee of Chongqing Medical University. After anesthetization using an aesthesia machine with isoflurane volatiles (RWD Life Science Co. Ltd., Shenzhen, China), the dorsal surface hairs of adult rats (male, 300 to 350 g) were shaved and a line cut (1 cm) was made from the back. Before treatment with various carbon clothes, iodoform and alcohol were used beforehand to avoid infection. Different square carbon clothes (*S* = 0.3cm^2^) were implanted under the full-thickness skin of the rats and kept away from the surgical wound. The surgical incision was then sutured, and to prevent infection, the incision was disinfected with iodophor every day. After 14 days, the rats were isoflurane-anesthetized and euthanized, and the skin at the site close to the carbon cloth was swiftly removed.

### Histological analysis

To evaluate the biocompatibility under full-thickness skin, hematoxylin–eosin staining was utilized to evaluate the inflammatory response in tissues. To investigate the impact of immunoreactions, antigen reaction, and cells dispersion in vivo, CD68, CD206 (i.e. MRC1), and 4′,6-diamidino-2-phenylindole immunofluorescence was used.

### CCK-8 and HeLa-RFP cultured with carbon cloth assay

The HeLa and HeLa-RFP cells were all cultured in DMEM with 1% penicillin-streptomycin solution and 10% fetal bovine serum under 37 °C in 5% CO_2_. The cytotoxicity of sensor surface was detected using a CCK-8 assay. The bare CC and CC/P3HT-N-MWCNTs were trim to 0.3 cm × 0.3 cm correspondingly, inserted in 96-well plates (5,000 HeLa cells each) for 6 h. The absorbance values were then measured at 450 nm with a spectrophotometer (Thermo Fisher Scientific–CN, USA) to assess the cytotoxicity of the HeLa cells. Meanwhile, HeLa-RFP cells were seeded on 2 kinds of carbon cloth in 96-well plates (5,000 HeLa-RFP cells each) and cultured for 6 h. Then, red fluorescence was used to observe the proliferation of HeLa-RFP cells.

### Calcein AM and PI fluorescence staining assay

A control group without carbon cloth, a bare carbon cloth (*S* = 0.3 cm^2^) experimental group, and a modified carbon cloth (*S* = 0.3 cm^2^) experimental group were set up in 96-well plates, respectively. A total of 400 μl of PC12 cell suspension was added to each, and after cross shaking, the cells were incubated in an incubator for 24 h. The working solution was configured according to Calcein AM (1,000 X): PI (1,000 X): detection buffer = 1:1:1,000. Ten microliters of working solution was added to each well, tinfoil-covered in a 96-well plate, and incubated at 37° for 30 min in the dark. The luminescence was then observed under a microscope.

## Data Availability

All data needed to evaluate the conclusions in the paper are present in the paper and Supplementary Materials. Additional data that are related to this paper may be requested from the authors.

## Supplementary Materials

Supplementary 1 Fig. S1. Photographs of modification process of CFME/P3HT-N-MWCNTs for mass production. Fig. S2. EDS spectrum of CF/P3HT-N-MWCNTs. Fig. S3. Electrochemical oxidation of AA and SEM characterization of different CFMEs. Fig. S4. Electrochemical oxidation of AA by glassy carbon electrodes. Fig. S5. Selectivity of CFME/P3HT-N-MWCNTs toward AA. Fig. S6. Photographs of brain slices and HT22 cells used in experiment. Fig. S7. The stability in brain slices of the CFME/P3HT-N-MWCNTs. Fig. S8. Electrochemical behavior of CFME/P3HT-N-MWCNTs in brain slices. Fig. S9. Electrochemical performance of glutamate and inhibitors used in the experiment. Fig. S10. Immunofluorescence images for carbon cloths implanted tissues. Fig. S11. Hematoxylin–eosin staining images of tissue responses following a 2-week implantation of carbon cloths. Fig. S12. The CCK-8 assay of Hela cells for carbon cloths. Fig. S13. Calcein AM and PI staining of PC12 cells on carbon cloth. Fig. S14. The fluorescence of Hela-RFP cells cultured on bare CC and CC/P3HT-N-MWCNTs. Fig. S15. Photographs of HT22 cells. Table S1. Comparison of the performance of AA biosensors modified with different membranes.

Supplementary 2 Movie S1. Water contact angle measurement for bare CC. Movie S2. Water contact angle measurement for CC/P3HT-N-MWCNTs.
