# Supplementary material for: A Novel Poly(3-hexylthiophene) Engineered Interface for Electrochemical Monitoring of Ascorbic Acid During the Occurrence of Glutamate-Induced Brain Cytotoxic Edemas
Source: Research (Wash D C). 2023 May 23;6:0149. doi: 10.34133/research.0149 (PMC10205589; doi:10.34133/research.0149)
Supplement: Supplementary 1 — Fig. S1. Photographs of modification process of CFME/P3HT-N-MWCNTs for mass production. Fig. S2. EDS spectrum of CF/P3HT-N-MWCNTs. Fig. S3. Electrochemical oxidation of AA and SEM characterization of different CFMEs. Fig. S4. Electrochemical oxidation of AA by glassy carbon electrodes. Fig. S5. Selectivity of CFME/P3HT-N-MWCNTs toward AA. Fig. S6. Photographs of brain slices and HT22 cells used in experiment. Fig. S7. The stability in brain slices of the CFME/P3HT-N-MWCNTs. Fig. S8. Electrochemical behavior of CFME/P3HT-N-MWCNTs in brain slices. Fig. S9. Electrochemical performance of glutamate and inhibitors used in the experiment. Fig. S10. Immunofluorescence images for carbon cloths implanted tissues. Fig. S11. Hematoxylin–eosin staining images of tissue responses following a 2-week implantation of carbon cloths. Fig. S12. The CCK-8 assay of Hela cells for carbon cloths. Fig. S13. Calcein AM and PI staining of PC12 cells on carbon cloth. Fig. S14. The fluorescence of Hela-RFP cells cultured on bare CC and CC/P3HT-N-MWCNTs. Fig. S15. Photographs of HT22 cells. Table S1. Comparison of the performance of AA biosensors modified with different membranes. [file research.0149.f1.pdf]

Supporting Information

A Novel Poly(3-hexylthiophene) Engineered Interface for Electrochemical Monitoring of Ascorbic Acid During the Occurrence of Glutamate-Induced Brain Cytotoxic Edemas

Zexuan Meng[1,†], Yuchan Zhang[1,†], Lu Yang[1,†], Shuang Zhao[2,3,†], Qiang Zhou[1,4], Jiajia Chen[1], Jiuxi Sui[1], Jian Wang[1], Lizhong Guo[1], Luyue Chang[1], Jialing He[2], Guixue Wang[2,3,*], Guangchao Zang[1,3,4,*]

[1]Institute of Life Science, and Laboratory of Tissue and Cell Biology, Lab Teaching & Management Center, Chongqing Medical University, Chongqing, 400016, China

[2]Key Laboratory for Biorheological Science and Technology of Ministry of Education, State and Local Joint Engineering Laboratory for Vascular Implants, Bioengineering College of Chongqing University, Chongqing, 400030, China

[3]Jinfeng Laboratory, Chongqing, 401329, China

[4]Department of Pathophysiology, Chongqing Medical University, Chongqing, China

†These authors contributed equally

*Address correspondence to: zangguangchao@cqmu.edu.cn; wanggx@cqu.edu.cn

Supporting Figures

Figure S1. Photographs of modification process of CFME/P3HT-N-MWCNTs for mass production.

Figure S2. EDS spectrum of CF/P3HT-N-MWCNTs.

Figure S3. Electrochemical oxidation of AA and SEM characterization of different CFME.

Figure S4. Electrochemical oxidation of AA by glassy carbon electrodes (GCEs).

Figure S5. Selectivity of CFME/P3HT-N-MWCNTs toward ascorbic acid.

Figure S6. Photographs of brain slices and HT22 cells used in experiment.

Figure S7. The stability in brain slices of the CFME/P3HT-N-MWCNTs.

Figure S8. Electrochemical behavior of CFME/ P3HT-N-MWCNTs in brain slices.

Figure S9. Electrochemical performance of glutamate and inhibitors used in the experiment.

Figure S10. Immunofluorescence images for carbon cloths implanted tissues.

Figure S11. HE staining images of tissue responses following a 2-week implantation of carbon cloths.

Figure S12. The CCK-8 assay of HeLa cells for carbon cloths.

Figure S13. Calcein AM and PI staining of PC12 cells on carbon cloth.

Figure S14. The fluorescence of HeLa-RFP cells cultured on bare CC and CC/P3HT-N-MWCNTs.

Figure S15. Photographs of HT22 cells.

Table S1. Comparison of the performance of AA biosensors modified with different membrane.

Reference

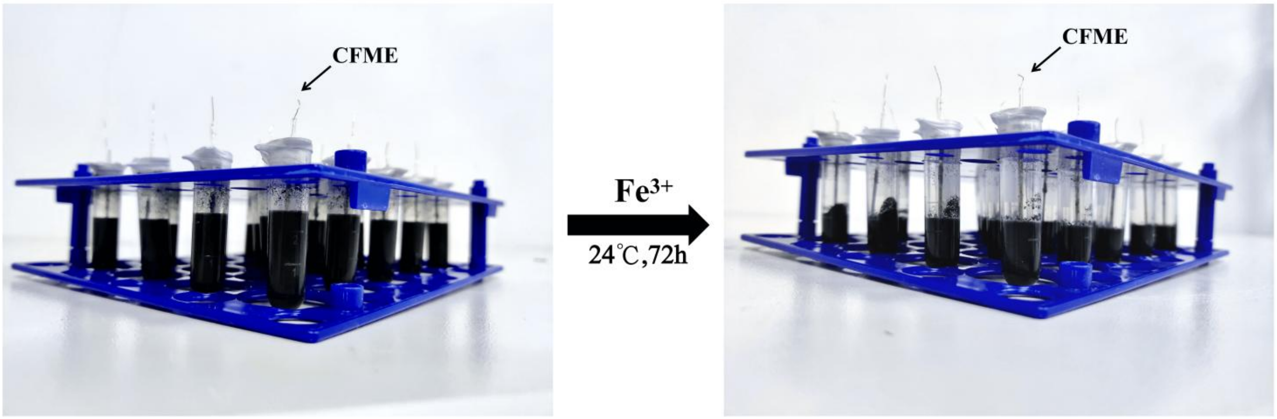

Figure S1. Photographs of modification process of CFME/P3HT-N-MWCNTs for mass production.

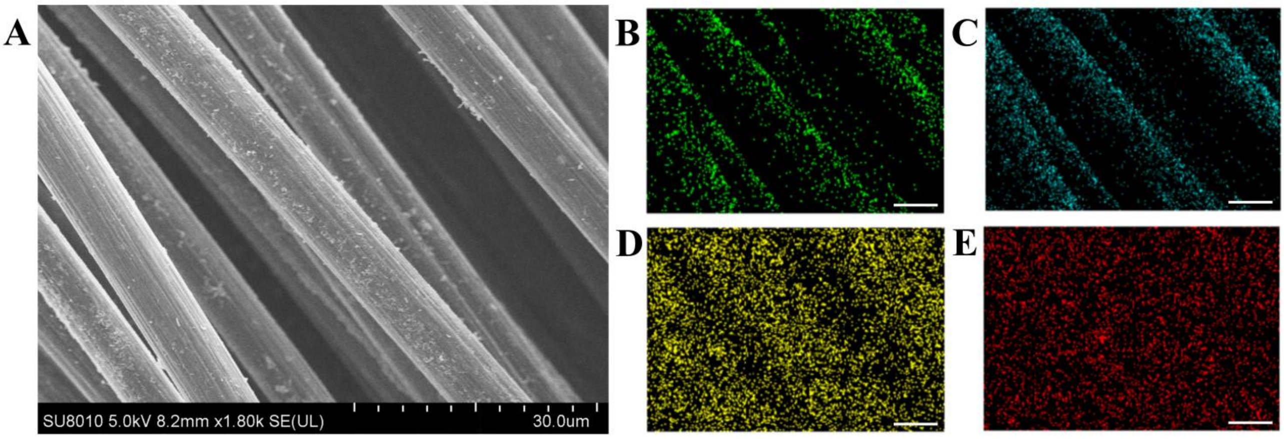

Figure S2. EDS spectrum of CF/P3HT-N-MWCNTs. SEM image(A) and diagram of the distribution of N elements(B), O elements(C), S elements(D), Fe elements(E) on CF. (scale bars=10μm).

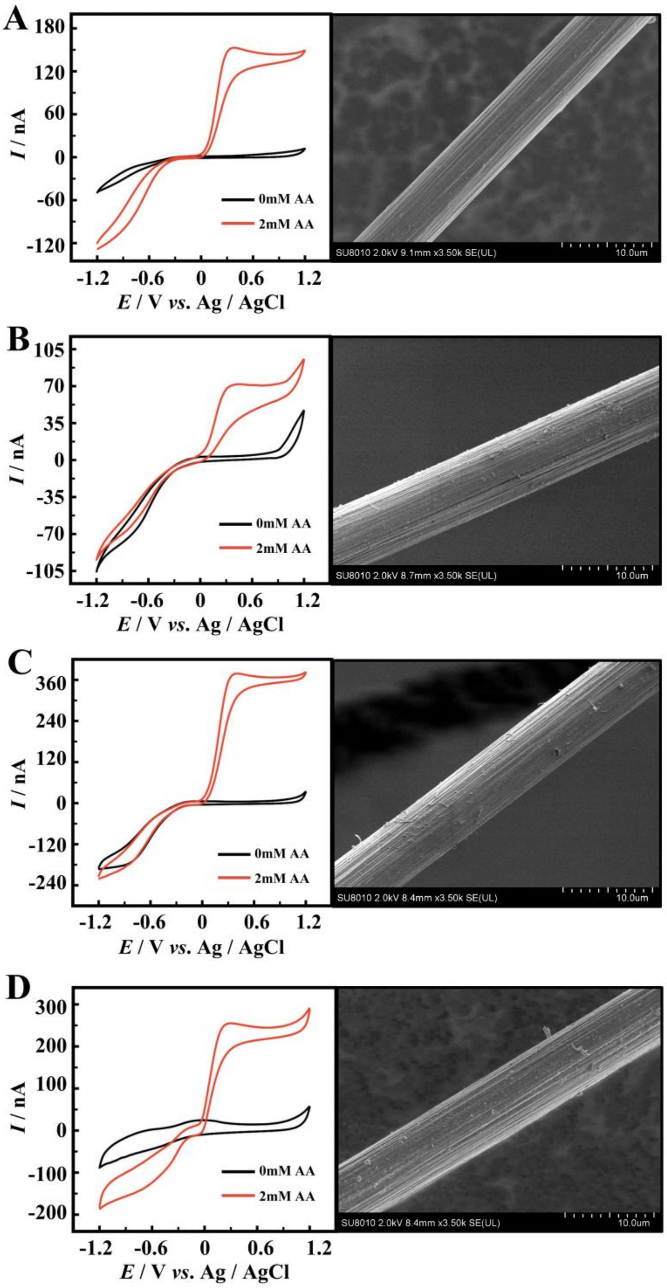

Figure S3. Electrochemical oxidation of AA and SEM characterization of different CFME. CVs and SEM images of AA (0 and 2 mM) at (A) bare CFME, (B) CFME/P3HT, (C) CFME/N-MWCNTs and (D) CFME/P3HT-N-MWCNTs. Scan rate:100 mV·s^{-1}.

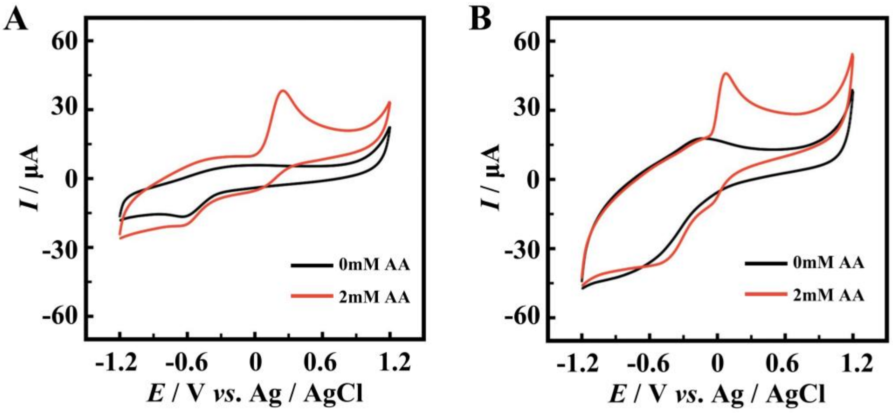

Figure S4. Electrochemical oxidation of AA by glassy carbon electrodes (GCEs). CVs of AA (0 and 2 mM) at (A) bare GCE and (B) GCE/P3HT-N-MWCNTs in aCSF. Scan rate:100 mV·s^{-1}.

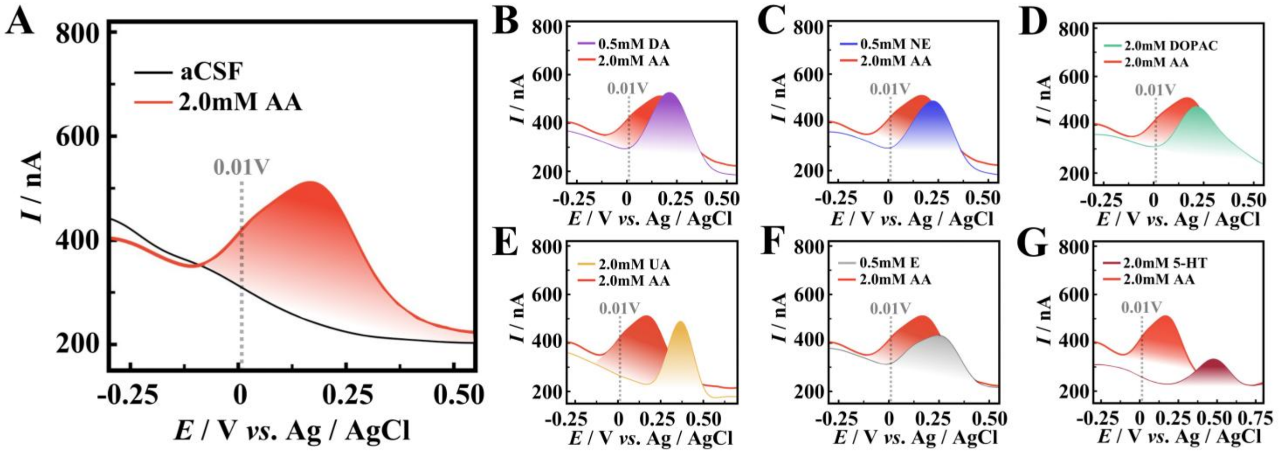

Figure S5. Selectivity of CFME/P3HT-N-MWCNTs toward ascorbic acid. DPVs images of 2.0 mM AA with (A) aCSF, (B) 0.5 mM DA, (C) 0.5 mM NE, (D) 2.0 mM DOPAC, (E) 2.0 mM UA, (F) 0.5 mM E and (G) 5-HT at CFME/P3HT-N-MWCNTs. The detection potential (0.01 V) is marked by dotted lines.

A B

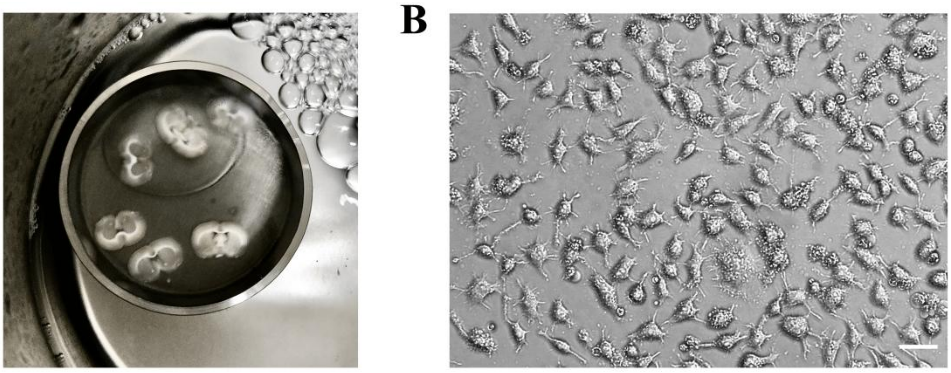

Figure S6. Photographs of brain slices and HT22 cells used in experiment. (A) brain slices, (B) HT22 cells (scale bar=50 μm).

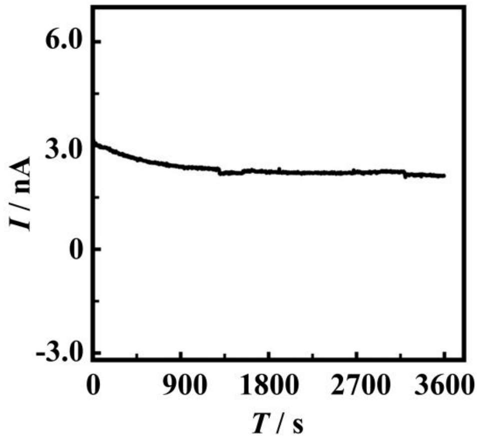

Figure S7. The stability in brain slices of the CFME/P3HT-N-MWCNTs. Amperometric response during 1 h implantation of the sensor into striatum of brain slices (n=3). Applied potential, +0.01 V vs. Ag/ AgCl.

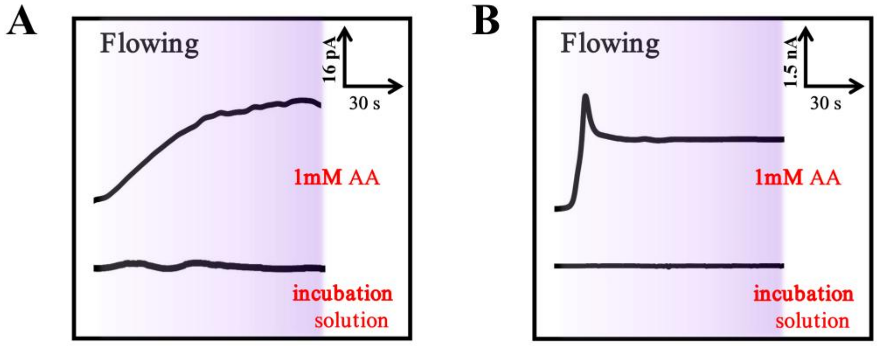

Figure S8. Electrochemical behavior of CFME/P3HT-N-MWCNTs in brain slices. (A) Amperometric response of AA in striatum of rat brain slices with CFME/P3HT-N-MWCNTs alone flowing infusion (2 mL/min for 60s) of 1 mM AA (upper), incubation solution (lower). (B) Amperometric response of CFME/P3HT-N-MWCNTs without rat brain slices alone flowing infusion (2 mL/min for 60 s) of 1 mM AA (upper), incubation solution (lower) (n=3). Applied potential, +0.01 V vs. Ag/ AgCl.

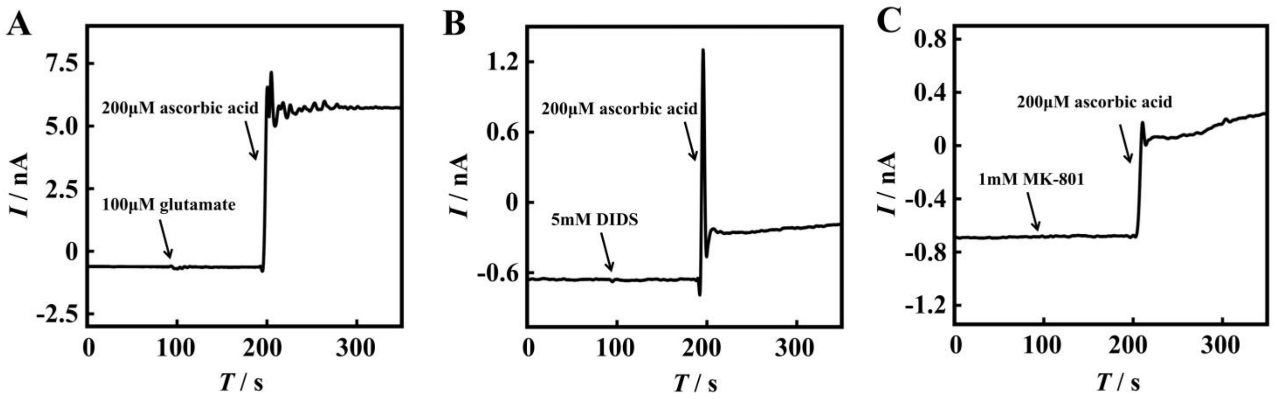

Figure S9. Electrochemical performance of glutamate and inhibitors used in the experiment. (A) 100 μM glutamate and 200 μM ascorbic acid, (B) 5 mM DIDS and 200 μM ascorbic acid, (C) 1 mM MK-801 and 200 μM ascorbic acid. Applied potential,+0.01 V vs. Ag/ AgCl.

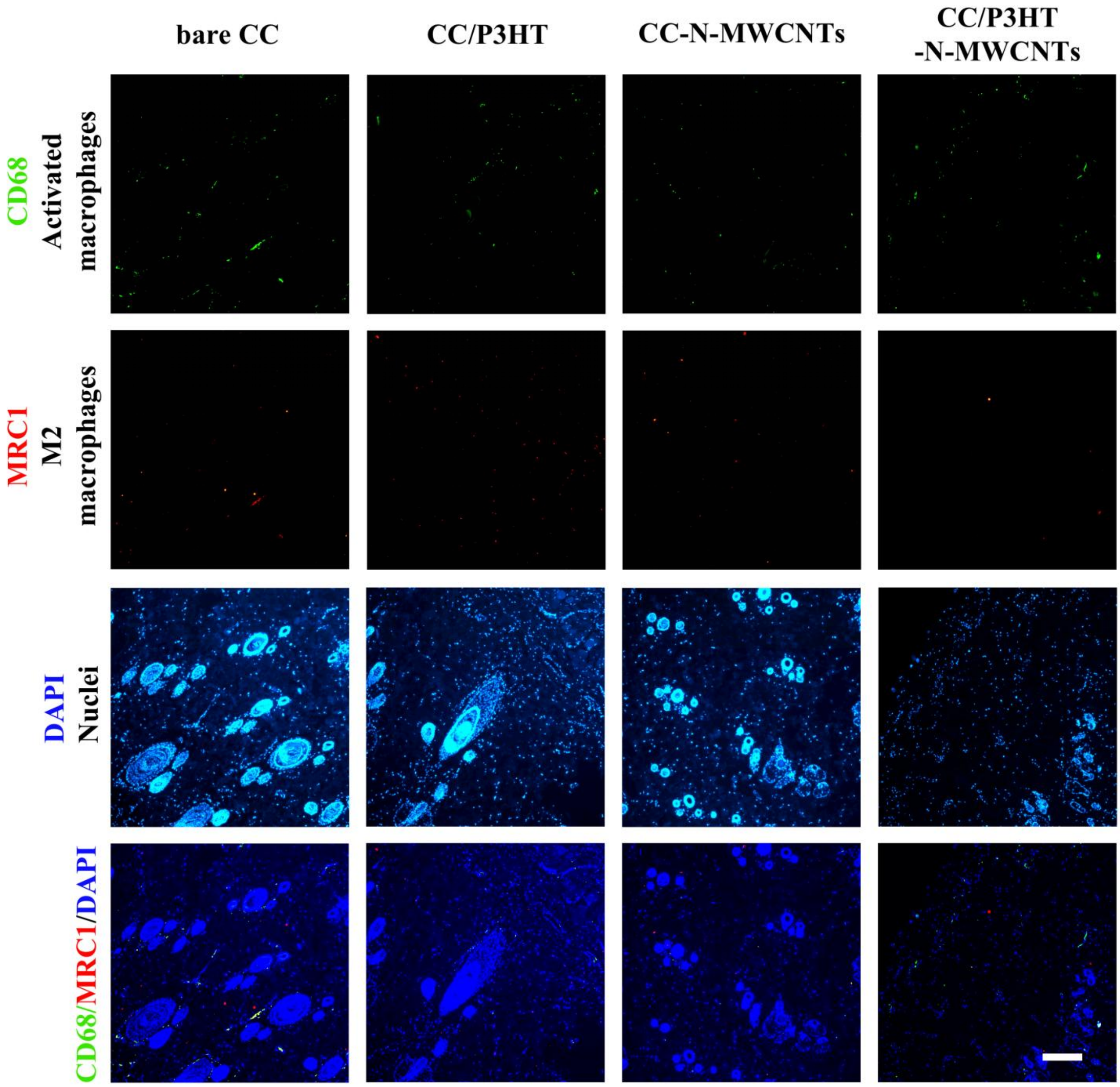

Figure S10. Immunofluorescence images for carbon cloths implanted tissues. Following a 2-week implantation of the bare CC, CC-N-MWCNTs, CC/P3HT and CC/P3HT-N-MWCNTs, from left to right. Representative immunohistochemistry images tagged with CD68 (green), MRC1(red) and DAPI (blue) , from top to bottom. (n=5, Scale bar=200 μm).

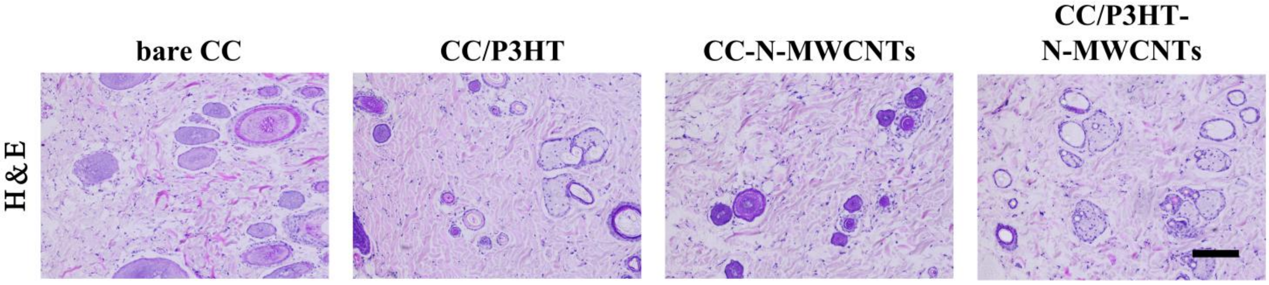

Figure S11. HE staining images of tissue responses following a 2-week implantation of carbon cloths. Bare CC, CC-N-MWCNTs, CC/P3HT and CC/P3HT-N-MWCNTs, from left to right. (n=5, Scale bar=300 μm).

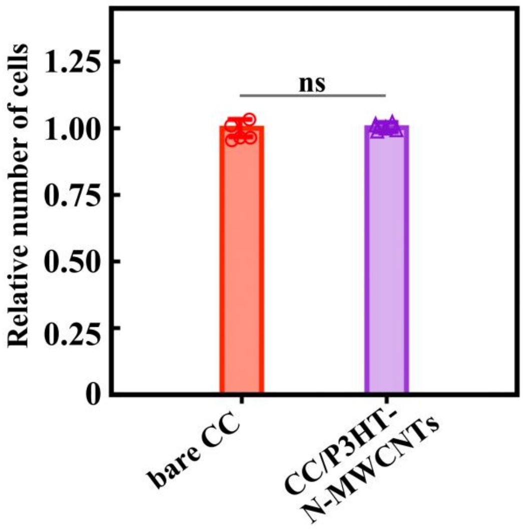

Figure S12. The CCK-8 assay of HeLa cells for carbon cloths. Bare CC (left) and CC/P3HT-N-MWCNTs (right) (n=5, ns: non-significant).

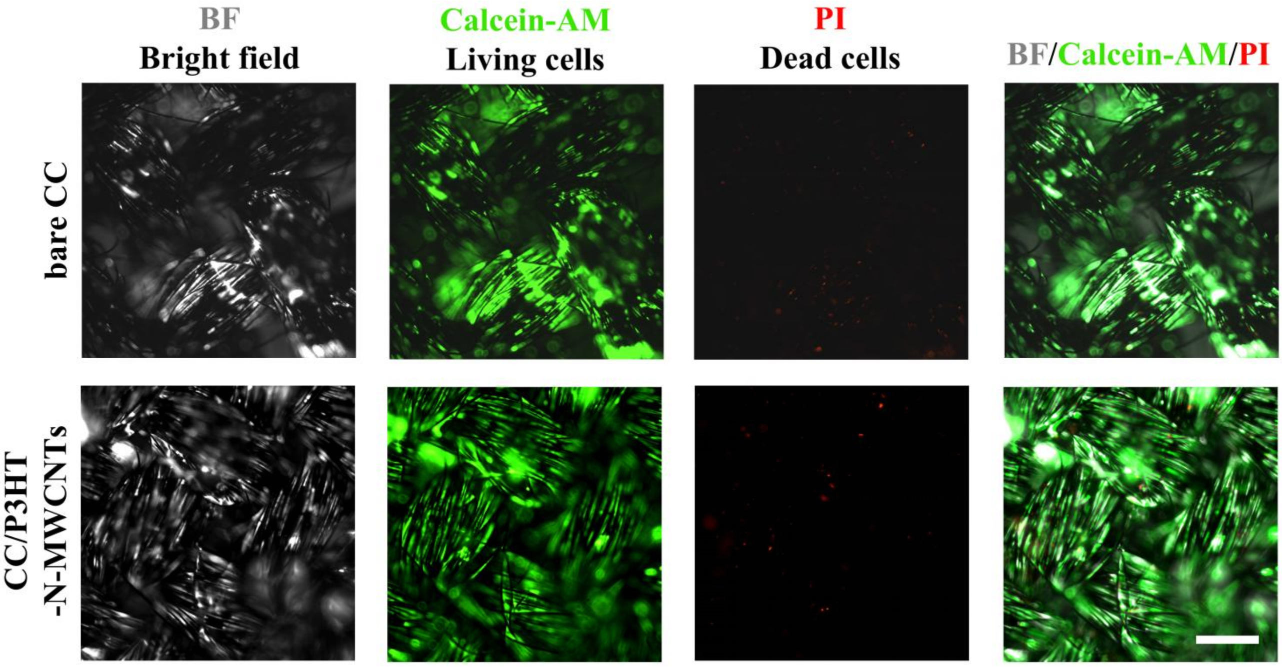

Figure S13. Calcein AM and PI staining of PC12 cells on carbon cloth. The upper images show the results of bare CC and the lower images show the results of CC/P3HT-N-MWCNTs (n=5, Scale bar=300μm).

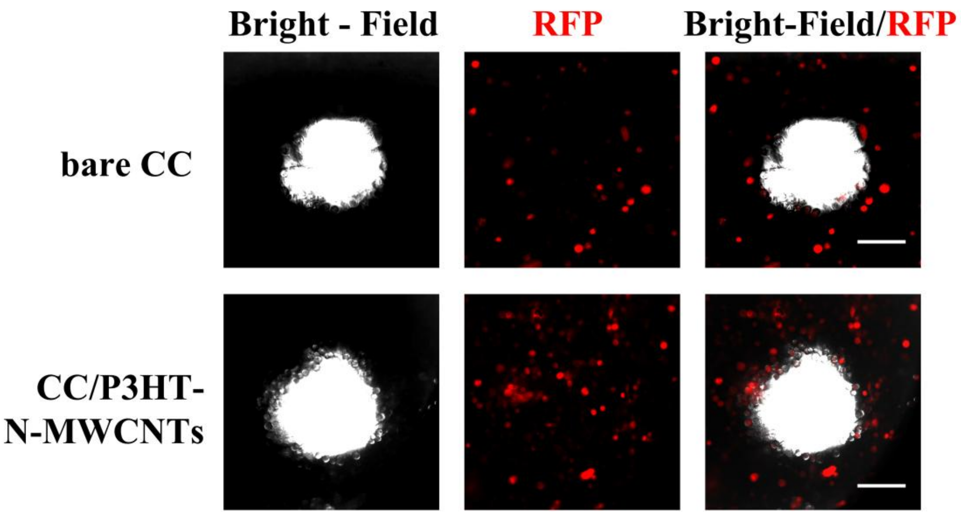

Figure S14. The fluorescence of HeLa-RFP cells cultured on bare CC and CC/P3HT-N-MWCNTs. The upper images show the results of bare CC and the lower images show the results of CC/P3HT-N-MWCNTs (n=5, scale bars=200 μm).

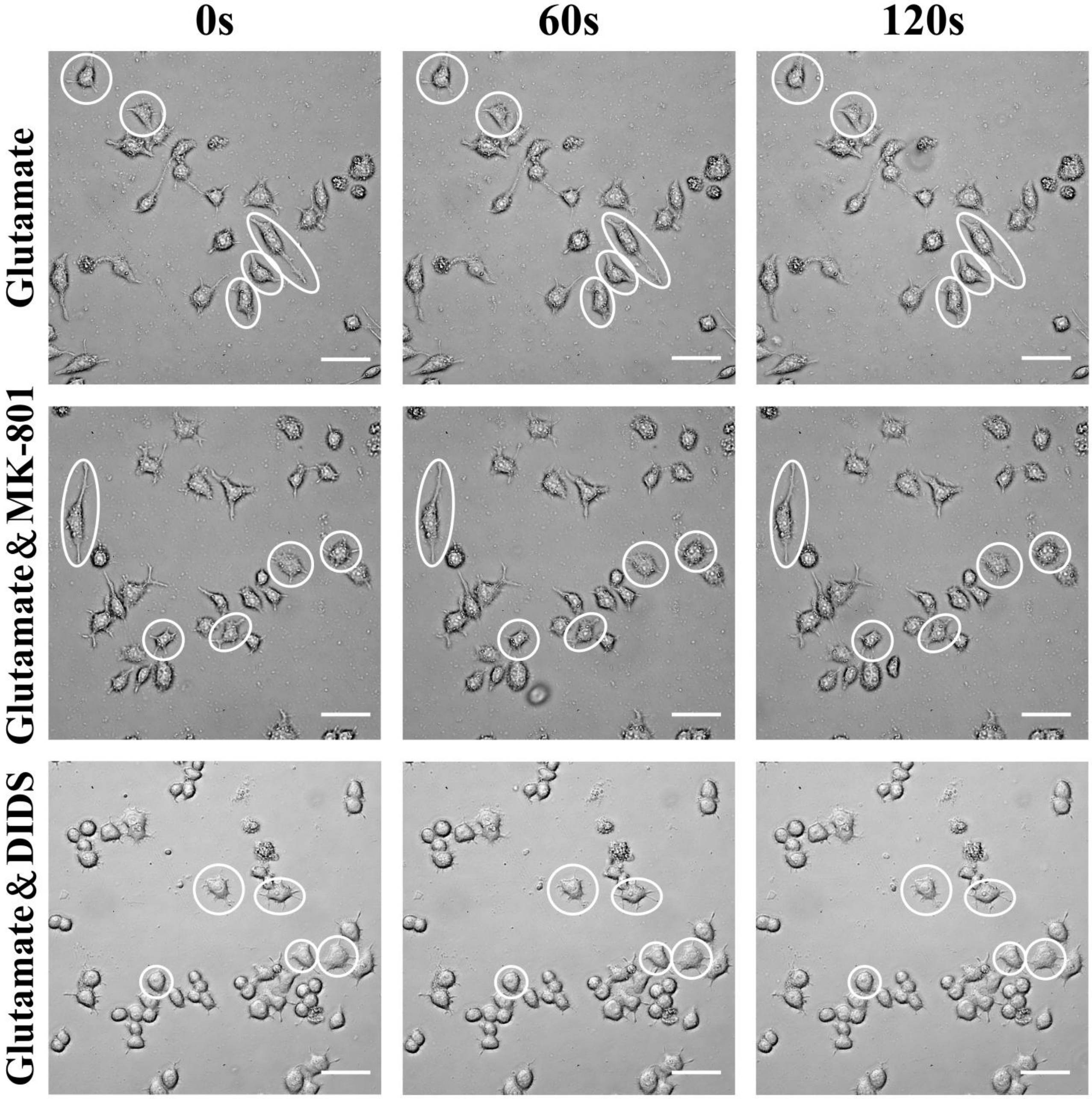

Figure S15. Photographs of HT22 cells. From top to bottom,cell is cultured by glutamate, glutamate & MK-801 and glutamate & DIDS. From left to right, time of processing 0 s, 60 s, 120 s (n=5, each for 5 cells, scale bars=100 μm).

Table S1. Comparison of the performance of AA biosensors modified with different membrane.

**Sensors**	**Method**	**Level**	**Linear Range / µM**	**Selectivity**	**Reference**
CFNT/RuOHCF	Amperometry	Cell	84-690	YES	[1]
CFE/Heptylamine-CSs	Amperometry	Brain	5-400	YES	[2]
GCE/PAY/MWCNTs	Amperometry	Urine	1-56	YES	[3]
CCE/MIP-MWCNTs	DPASV	Serum & CSF	0.057- 0.964	YES	[4]
GCE/p-TA/nano-Au	DPV	Serum & Urine	2.1-50.1	YES	[5]
Pt/PAAQ	SWV	Urine	112-1210	YES	[6]
GCE/p-PDB	DPV	Serum	0.8-70	YES	[7]
GCE/p-NALM	DPV	Urine	10-1000	YES	[8]
GCE/RGO-PEDOT	DPV	Serum	0.1-907	YES	[9]
CFME/P3HT-N-MWCNTs	Amperometry	Cell & Brain slice & Brain	10-400	YES	This work

**Sensors**	**Stability**	**Fouling Resistance**	**Biocompatibility**	**Reference**
CFNT / RuOHCF	NO	NO	NO	[1]
CFE/Heptylamine-CSs	YES	NO	NO	[2]
GCE/PAY/MWCNTs	YES	NO	NO	[3]
CCE/MIP-MWCNTs	YES	NO	NO	[4]
GCE/p-TA/nano-Au	YES	NO	NO	[5]
Pt/PAAQ	NO	NO	NO	[6]
GCE/p-PDB	YES	NO	NO	[7]
GCE/p-NALM	YES	NO	NO	[8]
GCE/RGO-PEDOT	YES	NO	NO	[9]
CFME/P3HT-N-MWCNTs	YES	YES	YES	This work

YES: This test was done and performance well. NO: This test was not done.

Reference

[1] Paixaeo, TRLC; Barbosa, LF; Carri, MT; Medeiros, MHG; Bertotti, M, Continuous monitoring of ascorbate transport through neuroblastoma cells with a ruthenium oxide hexacyanoferrate modified microelectrode. *Analyst* **2008,** *133* (11), 1605-1610. 10.1039/b805868g

[2] Jin, J; Ji, W; Li, L; Zhao, G; Wu, W; Wei, H; Ma, F; Jiang, Y; Mao, L, Electrochemically Probing Dynamics of Ascorbate during Cytotoxic Edema in Living Rat Brain. *Journal of the American Chemical Society* **2020,** *142* (45), 19012-19016. 10.1021/jacs.0c09011

[3] Kumar, SA; Wang, S-F; Yang, TCK; Yeh, C-T, Acid yellow 9 as a dispersing agent for carbon nanotubes: Preparation of redox polymer-carbon nanotube composite film and its sensing application towards ascorbic acid and dopamine. *Biosensors & Bioelectronics* **2010,** *25* (12), 2592-2597. 10.1016/j.bios.2010.04.024

[4] Prasad, BB; Jauhari, D; Tiwari, MP, A dual-template imprinted polymer-modified carbon ceramic electrode for ultra trace simultaneous analysis of ascorbic acid and dopamine. *Biosensors & Bioelectronics* **2013,** *50*, 19-27. 10.1016/j.bios.2013.05.062

[5] Wang, C; Yuan, R; Chai, Y; Zhang, Y; Hu, F; Zhang, M, Au-nanoclusters incorporated 3-amino-5-mercapto-1,2,4-triazole film modified electrode for the simultaneous determination of ascorbic acid, dopamine, uric acid and nitrite. *Biosensors & Bioelectronics* **2011,** *30* (1), 315-319. 10.1016/j.bios.2011.08.035

[6] Troiani, EdP; Faria, RC, Cathodically pretreated poly(1-aminoanthraquinone)-modified electrode for determination of ascorbic acid, dopamine, and uric acid. *Journal of Applied Electrochemistry* **2013,** *43* (9), 919-926. 10.1007/s10800-013-0577-7

[7] Hasanpour, F; Nekoeinia, M; Rashidi, H, Application of Pyrogallol Azo Derivative as a Mediator for Simultaneous Voltammetric Sensing of Ascorbic Acid, Epinephrine, Acetaminophen, and Tryptophan. *Ieee Sensors Journal* **2016,** *16* (22), 7992-7998. 10.1109/jsen.2016.2606422

[8] Kannan, A; Sivanesan, A; Kalaivani, G; Manivel, A; Sevvel, R, A highly selective and simultaneous determination of ascorbic acid, uric acid and nitrite based on a novel poly-N-acetyl-L-methionine (poly-NALM) thin film. *Rsc Advances* **2016,** *6* (99), 96898-96907. 10.1039/c6ra18440e

[9] Dinesh, B; Vilian, ATE; Kwak, CH; Huh, YS; Saraswathi, R; Han, Y-K, The facile and simple synthesis of poly(3,4ethylenedioxythiophene) anchored reduced graphene oxide nanocomposite for biochemical analysis. *Analytica Chimica Acta* **2019,** *1077*, 150-159. 10.1016/j.aca.2019.05.053
